# Gas hydrate dissociation linked to contemporary ocean warming in the southern hemisphere

**DOI:** 10.1038/s41467-020-17289-z

**Published:** 2020-07-29

**Authors:** Marcelo Ketzer, Daniel Praeg, Luiz F. Rodrigues, Adolpho Augustin, Maria A. G. Pivel, Mahboubeh Rahmati-Abkenar, Dennis J. Miller, Adriano R. Viana, José A. Cupertino

**Affiliations:** 1 Department of Biology and Environmental Science, Faculty of Health and Life Sciences Linnaeus University, SE-391 81 Kalmar, Sweden; 2 Géoazur, 250 rue Albert Einstein, 06560 Valbonne, France; 3 Petroleum and Natural Resources Institute, Pontificia Universidade Catolica do Rio Grande do Sul, Porto Alegre, CEP 91619-900 Brazil; 4 Instituto de Geociências, Universidade Federal do Rio Grande do Sul, Porto Alegre, CEP 91509-900 Brazil; 5 Petrobras Petroleo Brasileiro SA, Rio de Janeiro, CEP 20031-170 Brazil

**Keywords:** Carbon cycle, Environmental impact, Solid Earth sciences

## Abstract

Ocean warming related to climate change has been proposed to cause the dissociation of gas hydrate deposits and methane leakage on the seafloor. This process occurs in places where the edge of the gas hydrate stability zone in sediments meets the overlying warmer oceans in upper slope settings. Here we present new evidence based on the analysis of a large multi-disciplinary and multi-scale dataset from such a location in the western South Atlantic, which records massive gas release to the ocean. The results provide a unique opportunity to examine ocean-hydrate interactions over millennial and decadal scales, and the first evidence from the southern hemisphere for the effects of contemporary ocean warming on gas hydrate stability. Widespread hydrate dissociation results in a highly focused advective methane flux that is not fully accessible to anaerobic oxidation, challenging the assumption that it is mostly consumed by sulfate reduction before reaching the seafloor.

## Abstract (web summary)

Ocean warming could enable the release of methane related to hydrate dissociation from the ocean floor, a process thought to have triggered abrupt climate changes in Earth history. Here the authors detect this process in action, observing a massive release of methane from a site in the South Atlantic Ocean.

## Introduction

Submarine gas hydrates on continental margins store large quantities of methane (e.g., 0.5–12.7 × 10^21^ g ^1,2^), mainly produced by microbial degradation of organic matter, and argued to form a large capacitor able to regulate the Earth’s climate^3–6^. The global gas hydrate reservoir is particularly vulnerable to dissociation at its feather edge, i.e. where the top and base of the gas hydrate stability zone (GHSZ) intercept the seafloor as a consequence of decreasing pressure (depth) and increasing bottom water temperature. The feather edge typically lies in upper slope depths (300–600 m), where ocean warming and sea-level lowering are capable of reducing the volume of the GHSZ to drive sediment degassing^5^. Field observations^7–12^ and numerical modelling^13–16^ suggest that anthropogenic-related ocean warming may be causing hydrate dissociation in upper slope settings. However, the mass of methane reaching the atmosphere is forecast to have a minor impact on climate during this century^6,14,17,18^, in part owing to sulfate reduction in sediments^19^ and in part to the dissolution and oxidation of methane in the water column^20,21^. Clearly, it is important to improve our understanding of the long- to short-term dynamics of the upper limits of gas hydrate systems on upper continental slopes in relation to contemporary climate change, and the effectiveness of the sulfate reduction filter in preventing methane to reach the oceans.

Our multi-disciplinary and multi-scale investigation of a bottom simulating reflector (BSR) outcrop on the southern Brazilian margin allows an investigation of gas hydrate dynamics and ocean interactions over long- (millennial) to short- (decadal) scales and provides the first robust evidence from the southern hemisphere of hydrate destabilization related to contemporary climate change. Geochemical and geophysical data, including the first autonomous underwater vehicle (AUV)-borne sub-bottom profiles of a BSR outcrop, allow us to document a massive advective flux of methane through the feather edge of the GHSZ, resulting in the formation of an elongate pockmark field associated with hundreds of water column gas flares. The pockmarks record long-term degassing, possibly in response to stable post-glacial water temperatures, while the observed BSR outcrop is in thermodynamic disequilibrium with bottom water temperatures and the present-day edge of the GHSZ, consistent with ocean warming over several decades. Our results add to growing evidence that gas hydrate dissociation and sediment degassing related to contemporary ocean warming is a global phenomenon. The advective flux of methane through the feather edge of the GHSZ is three orders of magnitude greater than background diffusive flux and cannot be entirely consumed by anaerobic oxidation in the sediment, challenging the assumption that the sulfate filter prevents methane from reaching the seafloor. Nonetheless, gas bubbles are inferred to dissolve within 50 m of seafloor, consistent with methane oxidisation in the water column before reaching the atmosphere. Estimated methane leakage rates at the edge of the GHSZ on the Brazilian margin are lower than those in the northern hemisphere, and indicate that hydrate dissociation may be an important process in the global carbon cycle and the Earth’s climate in a long-term (e.g. 10^3^ years) perspective.

## Results and discussion

### Degassing along the edge of gas hydrate stability

**Fig. 1:**
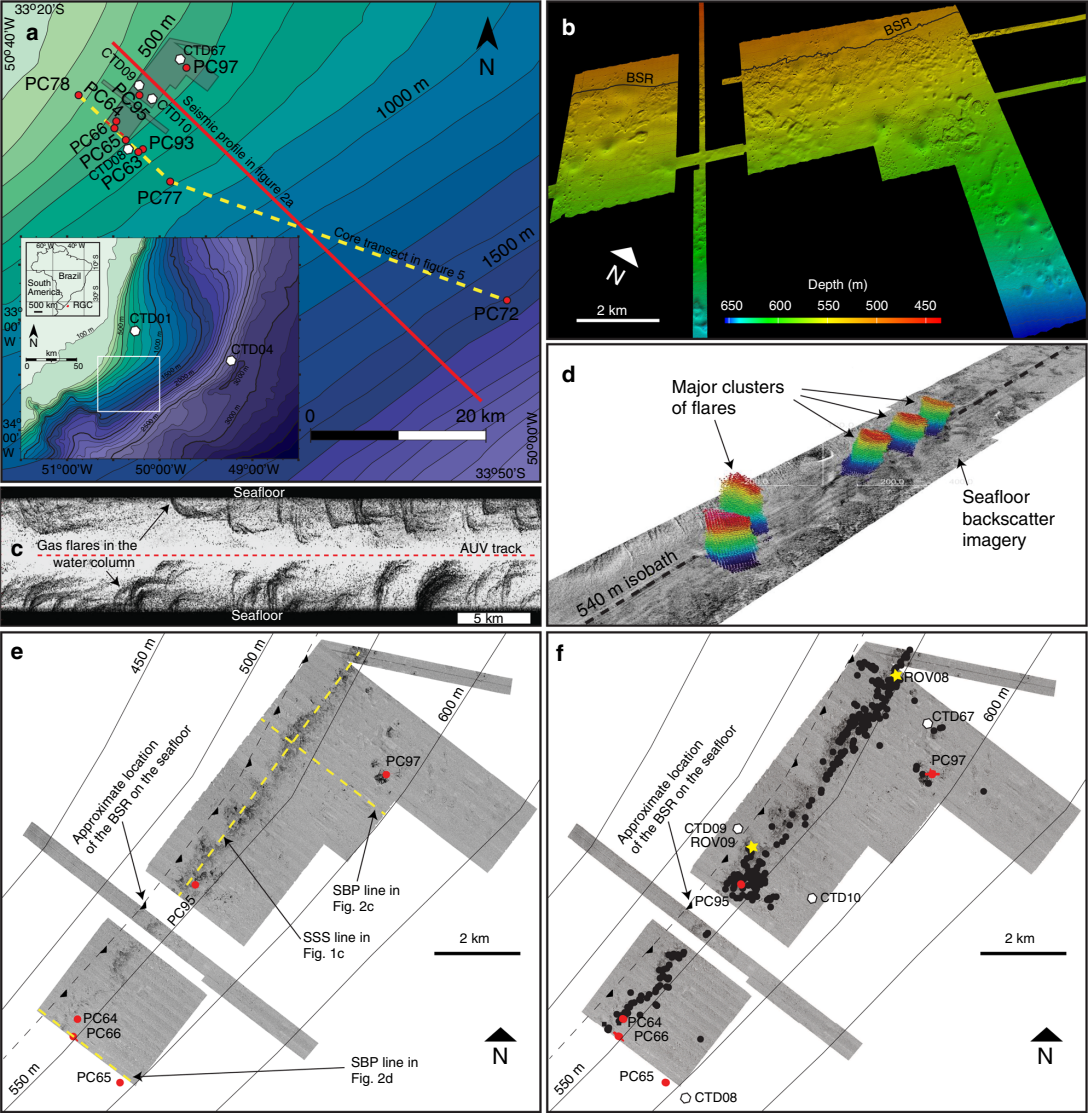
Location map and acoustic imagery of gas flares. **a** Location of the Rio Grande Cone (inset) and bathymetry of the study area showing the locations of piston cores (PC; red dots) and CTDs (white hexagons), the core transect shown in Fig. 5 (yellow dashed line), a regional seismic profile (red line), and the extent of autonomous underwater vehicle (AUV)-borne multibeam bathymetry (grey area); **b** a 3D perspective view of the seafloor from AUV-borne multibeam bathymetry showing the pockmark field downslope of the bottom simulating reflector (BSR) outcrop observed on seismic data; **c** raw water column imagery from AUV-mounted side-scan sonar showing dozens of gas flares rising from the seafloor; **d** water column and seafloor backscatter imagery from hull-mounted MBES showing major gas flare clusters ca. 50 m high (coloured features) aligned along the 540 m isobath; **e** AUV-mounted side-scan sonar (SSS) seafloor imagery showing high backscatter areas downslope of the BSR outcrop, consistent with the presence of carbonates within the pockmarks, and the location of sub-bottom profiles (SBP); **f** AUV-mounted side-scan sonar seafloor imagery showing the locations of gas flares (black dots), piston cores (red dots), conductivity, temperature, depth profiles (CTD; white hexagons), and bubble streams observed at seafloor using a remotely operated vehicle (ROV; yellow stars). Gas hydrate was recovered in piston cores PC66, PC95, and PC97.

The study area is located on the Rio Grande Cone (RGC), which consists of a 250,000 km^2^ and 7.5-km-thick, Barremian-Recent^22,23^ sediment depocenter in the southern Brazilian margin (Fig. 1a). A gas hydrate province was first recognised from a continuous, slope-crossing BSR observed over an area of at least 45,000 km^2^ in water depths of 500–3500 m^24,25^. Seafloor investigations have subsequently yielded samples of gas hydrates, authigenic carbonates, and chemosynthetic ecosystems indicative of gas venting from pockmarks fields in two areas, on the mid- and upper slopes^26–28^. Ship-borne multibeam bathymetric imagery show that the upper slope pockmark field lies in water depths of 520–660 m, near the upper limit of the GHSZ, and is elongated parallel to at least 12 km of the slope^26,29^ (Fig. 1b). Here we present additional evidence that this pockmark field is associated with gas venting to the water column (Fig. 1c, d), and show that it lies immediately downslope of the regional BSR outcrop on the seafloor (Fig. 1e, f).

**Fig. 2:**
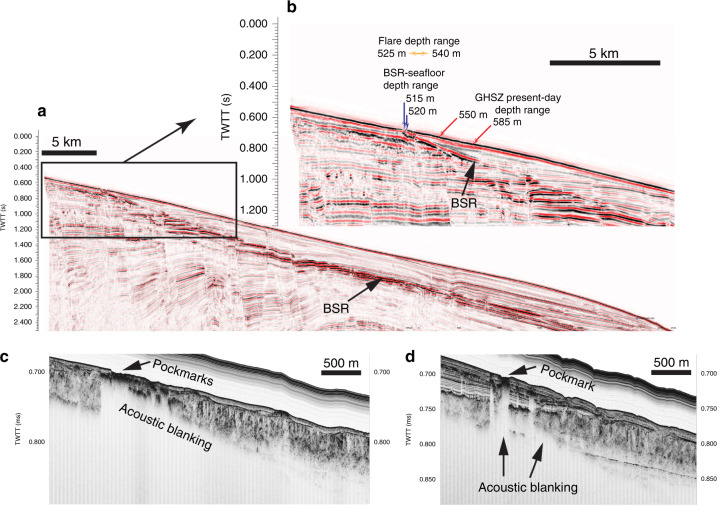
Regional seismic line and sub-bottom profiles. **a** Multichannel seismic profile showing the bottom simulating reflector (BSR) cross-cutting stratal reflections and rising upslope to intercept the seafloor; **b** detail of (**a**) showing the BSR outcropping at seafloor in water depths of 515–520 m; orange arrows indicate the depth range of most of the flares in the area (525–540 m), red arrows indicate the present-day depth range of the gas hydrate stability zone (GHSZ) calculated from bottom water temperatures (550–585 m); **c**, **d** Autonomous underwater vehicle (AUV)-mounted sub-bottom profiles showing acoustic blanking below the pockmark field and the BSR, consistent with free gas rising from BSR depth towards seafloor. The pockmark field is located at 520–560 m of water depth. See Fig. 1 for locations of the profiles.

**Fig. 3:**
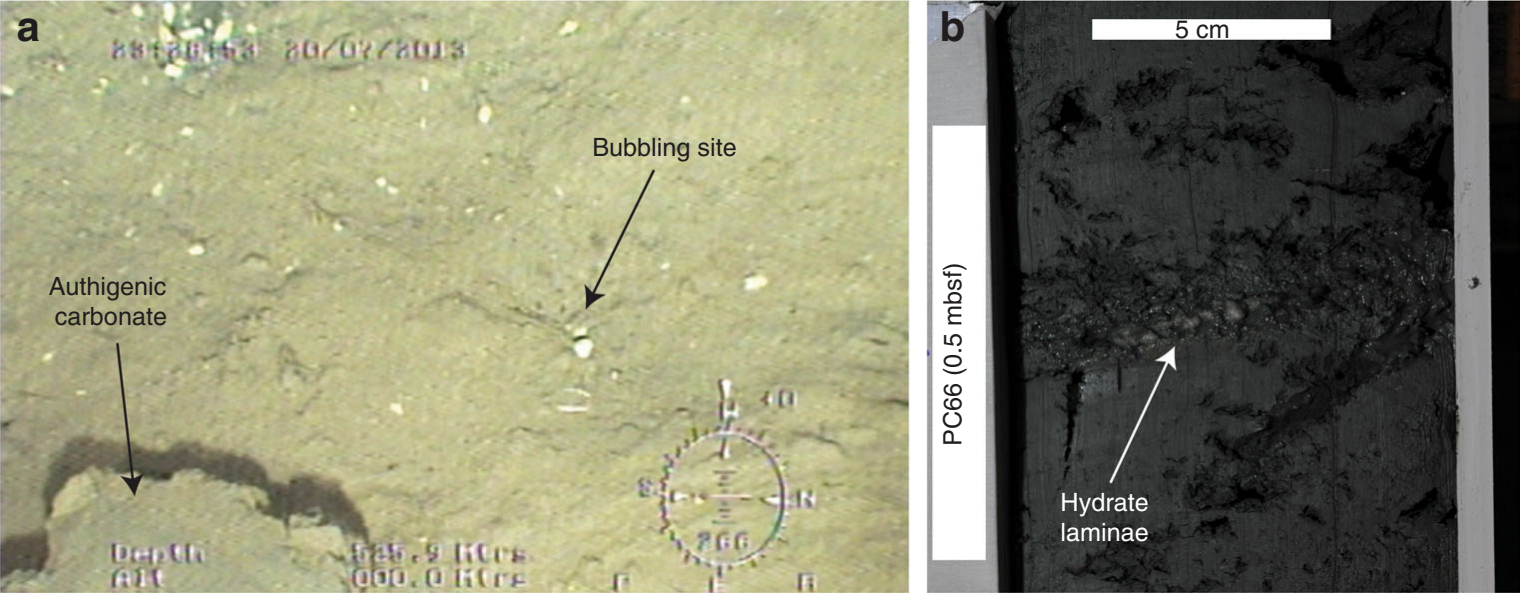
Seafloor gas bubble stream and gas hydrate in sediment. **a** Bubbling site and authigenic carbonate block on the seafloor in a pockmark obtained by inspection with a remotely operated vehicle at the ROV08 flare site; and **b** thin hydrate laminae in dark grey massive mud obtained from piston core PC66 (see Fig. 1 for location).

**Fig. 4:**
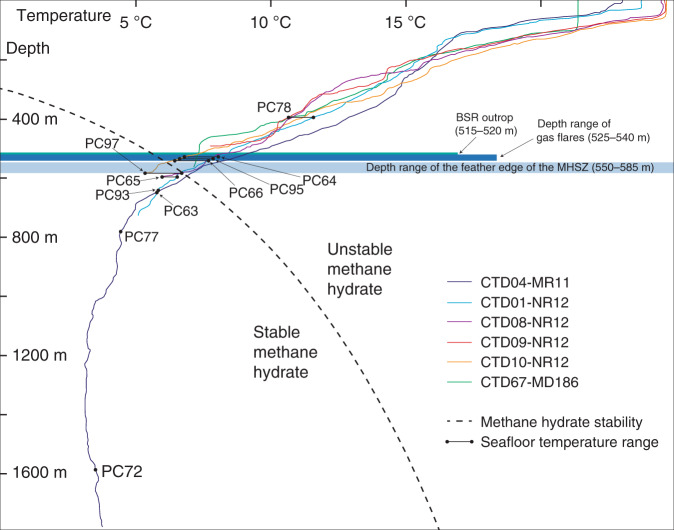
Depth versus temperature diagram. Illustration showing the temperature profiles from conductivity, temperature, depth (CTD) casts, the location of piston cores, the bottom simulating reflector (BSR) outcrop, depth range of gas flares and the depth range of the feather edge of the methane hydrate stability zone. The stability of methane hydrate was calculated assuming pure methane in seawater^34^.

Seismic reflection profiles across the RGC upper slope (Fig. 2a, b) show that the regional BSR intercepts the seafloor at ca. 515–520 m water depth, at the upper limit of the pockmark field. The BSR outcrop is observed both on conventional multichannel seismic profiles (peak frequencies 10^1^ Hz) and, to our knowledge for the first time, on sub-bottom profiles (SBP) acquired 40 m from seafloor using an AUV (peak frequencies 10^3^ Hz). The AUV profiles show strong acoustic blanking, both at depths corresponding to the BSR, and as vertical columns rising above the BSR (Fig. 2c, d). Blanking on high frequency data is attributable to attenuation of the acoustic signal by reverberation and scattering due to the presence of gas bubbles in sediment pores^30–32^. The blanking features on SBP imagery are therefore consistent with the presence of free gas both beneath the BSR and rising through the thin (10s m) GHSZ, beneath the pockmark field. At the seafloor, AUV-borne multibeam imagery (Fig. 1b) reveals hundreds of high backscatter depressions, up to 600 m wide and 3–8 m deep, and thousands of smaller unit pockmarks^33^. High backscatter signatures are consistent with the presence of authigenic carbonates, as observed at the seafloor over wide areas using a remotely operated vehicle (ROV; Fig. 3a). Raw side-scan sonar images reveal 394 gas flares rising up to 50 m into the water column, from seafloor depths of 525–540 m, which can also be observed in ship-borne multibeam imagery (Fig. 1c, d). The flares are, therefore, located between the BSR outcrop at 515–520 m and the edge of GHSZ at 550–585 m calculated using water column temperature measurements obtained locally in the pockmark field (Figs. 2b and 4; Supplementary Data 1) and the equilibrium equation for pure methane hydrate in seawater^34^.

Nine giant Calypso-type piston cores (20–40 m long) were obtained within the pockmark field and along a transect crossing it in water depths of 397–1588 m (Fig. 1a) and show the presence of massive and laminated, bioturbated, dark olive and dark greenish grey, rarely dark grey, muddy sediments (Supplementary Fig. 1) containing authigenic carbonate nodules (centimetres in diameter). Gas hydrate was recovered in three piston cores within the pockmark field (PC95, PC66, and PC97) in water depths of 550–585 m, as mm−cm-thick laminae oriented parallel to sediment layering, at depths of up to 8 mbsf (Fig. 3b).

### Advective vs diffusive gas fluxes

Evidence from ROV observations and cores allow us to quantify both the advective flux of methane to the ocean through the edge of the GHSZ and the broader diffusive flux of methane towards seafloor across the upper slope. Within the area of gas flares, ROV seafloor observations at two sites (Fig. 1f) indicate that acoustically observed flares are formed by 5–6 individual bubble streams, which flow intermittently, varying from virtually zero to up to three bubbles per second in a time span of less than a minute (Fig. 3a, Supplementary Movie 1). Bubbles are estimated to be ca. 1 cm in diameter based on observations during sampling of a single bubble stream at each site (see “Methods”), but may show a high variability in diameter, even in the same cluster^35^, and commonly between 0.2 and 0.5 cm as observed in other seep sites around the world^35–37^. Observations of the two sites in the RGC over longer periods (up to 1 h) show a lower bubbling rate of 0.023–0.106 bubbles per second (44–200 cm^3^ h^−1^) and mass transfer rates of 0.5 × 10^–3^ g s^−1^ and 2.3 × 10^–3^ g s^−1^ (for bubble of 1 cm in diameter; see Supplementary Note 1 for mass transfer rates and methane fluxes calculated for different bubble sizes and bubbling rates). Assuming these rates apply to all 394 flares mapped in the area, and that each flare contains five bubble streams (an assumption that is in agreement with observations of other flares offshore Svalbard^38^, Pakistan^35^, and Cascadia margin^39^), the total methane advective mass flux is 25.2–115.9 mmol cm^−2^ year^−1^, and the total methane mass transfer rate from sediments to the ocean is 31.3–144 Mg year^−1^.

**Fig. 5:**
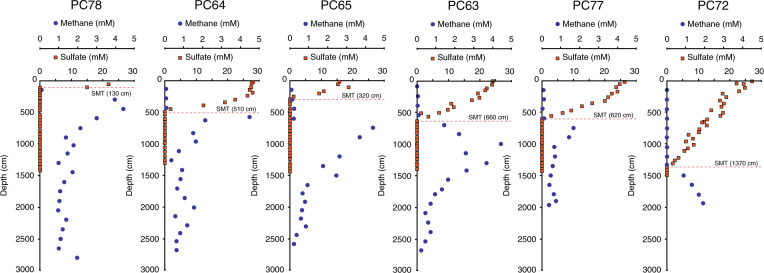
Methane and sulfate concentrations in pore waters. A transect across the edge of the hydrate stability zone with six piston cores (PC) showing methane and sulfate concentration in pore waters (location in Fig. 1). The depth of the sulfate−methane transition (SMT) is indicated in each profile with a dashed orange line, drawn at the depth of no sulfate.

A transect of six piston cores across the edge of the GHSZ (Fig. 1a) provides an understanding of methane distribution below the seafloor and estimates of its diffusion rates, based on measurements of sulfate concentrations in pore waters^40^ (Fig. 5; Supplementary Data 2). Along the transect, the depth of the maximum methane concentration increases downslope from the hydrate-free area into the GHSZ, while the depth of the sulfate methane transition (SMT) is the shallowest (1.3 m) in the hydrate-free area and progressively deepens downslope within the thickening hydrate stability zone (from 3.2 to 13.7 m; Fig. 5). The increasing depth of the SMT and maximum methane concentration, and related smaller methane flux within the thickening GHSZ are attributed to a combination of the uptake of methane via hydrate precipitation^41^, smaller in situ methane yield caused by more limited access of methanogenic microbes to young and labile organic matter in deeper SMT settings^42^, and possibly lower temperatures in deeper bottom waters.

Our observations show that sulfate reduction is not able to oxidise the massive advective flux of methane in the flares (25.2–115.9 mmol cm^−2^ year^−1^), which is up to three orders of magnitude higher than the maximum calculated diffusive methane mass flux (35.6 × 10^–3^ mmol cm^−2^ year^−1^), and up to three orders of magnitude higher than the maximum diffusive flux recognised in the oceans^42^. It is interesting to note that this massive advective flux bypasses the local sulfate reduction zone without changing the local SMT depth (5.1 m at PC64; Fig. 5), indicating it is very localised. This result implies that the advective flux of methane is not entirely accessible to sulfate reduction^6,43^. The mechanisms by which a focused methane flow migrates through the sediment to bypass the sulfate reduction filter remain poorly understood, but recent numerical modelling has shown that rapid gas hydrate dissociation in sediments at shallow depths (<14 m) below seafloor may create hydraulic fractures that focus methane flow^44^, producing a flux that cannot be entirely consumed by sulfate reduction^45^. Our results are therefore consistent with a highly focused massive advective flux of methane within the pockmark field that is bypassing the sulfate reduction filter to form gas flares. This finding challenges the assumption that the majority of the methane released by hydrate dissociation in upper slope settings will be consumed anaerobically before reaching the seafloor^10,46^.

### Origin of gas

**Fig. 6:**
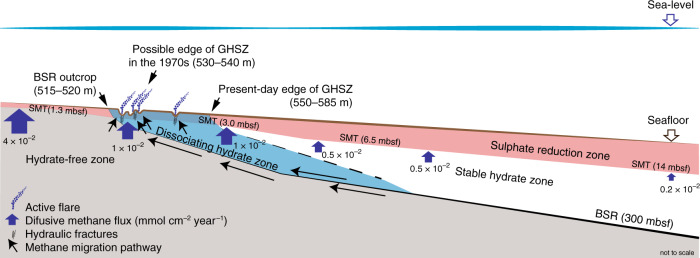
Section across the edge of the hydrate stability zone. Schematic section across the upper slope of the Rio Grande cone, showing the location of active gas flares, together with estimates of methane diffusive flux (mmol cm^−2^ yr^−1^). Blue arrows indicate possible methane migration pathways in the hydrate-free, dissociating hydrate, and stable hydrate zones. Note the diminishing diffusive methane flux and deepening of the sulfate−methane transition (SMT) downslope towards a thickening gas hydrate stability zone (GHSZ) indicated by the bottom simulating reflector (BSR).

Methane is the dominant gas present in all bubble, hydrate, and pore water samples (Supplementary Data 3). Carbon stable isotopic analyses reveal a wide range of δ^13^C values (Supplementary Data 4) compatible with a biogenic origin (<−50‰^47^). Methane sampled within the pockmark field has no detectable ^14^C isotope, indicating that it is sourced from fossil carbon (>43,500 years B.P.). The similar δ^13^C values in gas bubbles and gas hydrate, together with the absence of ^14^C, suggest that in situ dissociation of hydrate trapping methane with fossil carbon is the likely source of methane in the flares. However, a noticeable enrichment in ^13^C in methane in pore waters from the pockmark field relative to hydrate suggests that in situ hydrate dissociation is not the sole source of methane. Deeper biogenic methane is commonly enriched in the ^13^C isotope^48^. Acoustic blanking beneath the pockmarks indicating free gas rising from beneath the BSR strongly supports the idea that biogenic methane is also being sourced from or below the base of the stability zone (Fig. 2c, d). In either case, methane may flow laterally underneath the base of the GHSZ^49^ to mix with gas in sediment pores and vent to the seafloor (Fig. 6). In contrast, downslope of the flares area pore water methane is found to have highly depleted in δ^13^C values as well as modern ^14^C (e.g. PC93; Supplementary Data 4), indicating in situ methanogenesis with little or no mixing with methane from advective sources.

### Degassing in response to stability zone dynamics

Methane release to the water column on the upper RGC is interpreted to be taking place by advective flow through the feather edge of the GHSZ from sources at or beneath its base, most likely via a sub-vertical system of fractures that, over time, have facilitated the formation of a pockmark field in water depths of 520–660 m (Fig. 1b). The presence of carbonate concretions resulting from the anaerobic oxidation of methane^26^ implies high gas flux over timescales of at least thousands of years at this location^29^. The striking parallelism of the pockmark field with the adjacent BSR outcrop in water depths of 515–520 m raises the question of whether gas venting could record the response of the feather edge of the GHSZ to changing oceanographic conditions since the last glacial maximum (LGM, ca. 20 ka BP).

Globally, ocean warming since the LGM has counteracted the effect of rising sea levels (pressure increase) on gas hydrate stability, notably on upper continental slopes where the influx of warmer water resulted in downslope retreat of its feather edge^15,50^. Post-glacial warming is argued to have driven widespread hydrate dissociation and degassing along continental margins, leading to the formation of a gas hydrate-depleted zone on upper slopes^51^. Such a natural, long-term depletion process may have diminished the quantity of gas hydrate available for dissociation related to short-term anthropogenic ocean warming^15^. On the southern Brazilian margin, in contrast, it has been hypothesised that elevation of the permanent thermocline during sea-level rise resulted in a post-glacial cooling of the upper slope that suppressed hydrate dissociation and methane release^52^. The proposed mechanism was suggested to be relevant to the gas hydrate system of the RGC^52^, where the base of the permanent thermocline lies in depths of 500–700 m and so contains the upper limit of the GHSZ^29^. We note that long-term post-glacial cooling would have favoured gas accumulation and so could account for the presence of a well-developed BSR outcrop, making the southern Brazilian margin a potential modern analogue to LGM margins. However, constraints on deglacial temperature changes in the water column on the upper RGC slope record temperature increases of 3.5 °C in bottom waters for the interval 18–10 ka BP, with a possible peak of 6.6 °C at ca. 14 ka BP^53^. The latter value is comparable to present-day bottom water temperatures, suggesting a long-term (latest 14 ka) stabilisation of temperatures that would have maintained the edge of the GHSZ near its present-day depth. Long-term stability of the GHSZ could in part account for a well-developed BSR outcrop in the RGC.

Over shorter timescales, it is of interest to note that while the narrow (<3 km wide) pockmark field lies downslope of the BSR outcrop in water depths of ca. 515–520 m, present-day water column temperatures within the same area indicate the upper limit of GHSZ to lie at deeper waters (550–585 m; Fig. 2b). Historical water temperature measurements from the South Atlantic provide evidence of a warming of the Antarctic Intermediate Waters (AAIW). This water mass is formed near the Antarctic Circumpolar Current, flows at >500 m water depth at the RGC, and is characterized by a salinity minimum and dissolved oxygen maximum^54^. The warming of the AAIW can be traced back at least to the 1970s, at rates of 0.01–0.02 °C year^−1 ^^55^, associated with contemporary climate change. These rates would position the edge of the GHSZ in the 1970s at 530–540 m water depth, within the depth range of most present-day gas flares on the RGC (Fig. 6). We suggest that recent ocean warming has unbalanced the edge of a long-term, stable gas hydrate system, which is in thermodynamic disequilibrium and undergoing dissociation downslope of the BSR outcrop (Fig. 6). Evidence of such a process has previously been recognised in the northern hemisphere on the US North Atlantic coast^8^ and in the Arctic on the western Svalbard margin^9^, but this is the first time that it is identified in the southern hemisphere. It is interesting to note that the methane emission rates estimated for the 12-km-long studied RGC section (0.16 × 10^6^−0.75 × 10^6^ mol year^−1^ km^−1^) are smaller than those of the western Svalbard margin^38,56,57^, suggesting a methane emission range of 10^5^−10^7^ mol year^−1^ km^−1^ for such settings.

## Methods

### Acoustic surveys

2D multichannel seismic data, acquired for hydrocarbon exploration and interpreted using IHS Kingdom Suite software, with a dominant frequency of 40 Hz, was used to map the BSR outcrop in the continental slope. AUV-borne acoustic data were obtained using a C-Surveyor II Autonomous Underwater Vehicle flying at 40 m above the seafloor and the Rig Supporter vessel. Seafloor morphology and imagery data were obtained with a Simrad EM 2000 multibeam echo sounder (operating frequency of 200 kHz) and a dual frequency (120–410 kHz) EdgeTech Side Scan Sonar (2200-M). Data were processed and visualised with IVS-3D Fledermaus and Sonar Wizz software. Sub-bottom profiler lines were acquired using an EdgeTech DW106 SBP (Chirp 1–6 kHz, central frequency 3.5 kHz), and lines were processed and visualised using the IHS Kingdom software. The major clusters of gas flares shown in Fig. 1d were located using the water column backscatter data obtained from the hull-mounted multibeam echo sounder installed onboard the Rig Supporter vessel. The water column backscatter data, which are highly sensitive to the presence of gas bubbles in water and, therefore, can be used to detect flares^58^, were integrated with the seafloor backscatter data using the IVS-3D Fledermaus software. Individual gas flares shown in Fig. 1f were located using the high-resolution water column data obtained from the AUV-mounted multibeam echo sounder and side-scan sonar systems. Two flares (marked in Fig. 1) were selected for ROV investigations. The ROV front sonar was used to reach the flares.

### Water column temperature measurements

A total of nine temperature and salinity profiles were obtained in the RGC using Sea-Bird Electronics CTD (Conductivity, Temperature, Depth) models SBE 19plus, SBE 9plus, and a RBR XR-620 CTD instrument. Water masses were identified based on their thermohaline indexes obtained by CTD data.

### Flare inspection and gas sampling procedure

Two ROV dives at flare sites previously identified with side-scan sonar images and sub-bottom profiles were performed using a Mohican Inspection Class Remotely Operated Vehicle (ROV) capable of operating in depths of up to 2000 m. The ROV was installed onboard the research vessel *Marion Dufresne* and was equipped with two manipulators and three inspection-quality video cameras. The positioning of the ROV was determined by a high precision acoustic positioning system (HiPAP). The location of bubbling sites was performed using the ROV’s front sonar, and bubbling gas was collected using a device consisting of a 300 mL stainless-steel bottle with vacuum, coupled to a ball valve and a transparent, acrylic funnel with markings to estimate bubble size. The device was positioned on top of the bubble stream and the time elapsed to fill a certain volume in the funnel was used to estimate the gas seepage rate. The valve was then opened and closed by the ROV’s manipulators to collect and store the gas (and water) in the bottle for further analyses. Gas seepage was also quantified by counting the number of bubbles per time, and a measured bubble diameter of 1 cm. The amount of methane *n* (moles) contained in a single bubble was determined by the gas law: (*n* = *PV*/*ZRT*), where *Z* is the compressibility of methane (0.8702), *P* is pressure (5.294 MPa at 525 m of water depth), *V* is the bubble volume, *R* is the universal gas constant, and *T* the ambient water temperature (8.5 °C). The compressibility factor of methane was calculated by the Peng−Robinson equation of state^59^. The annual mass transfer of methane for the 394 flares was calculated assuming an average of five bubble streams per flare in a total area of ca. 8000 m^2^. The calculations assume no temporal variability of advective flux on timescales longer than 1 h, no seasonal variations in the flux as observed in other seep sites^60–62^, a uniform rate of discharge at all 394 flare sites, and a single bubble diameter of 1 cm for all bubbles (see Supplementary Note 1 for mass transfer rates and methane fluxes calculated for different bubble sizes and bubbling rates).

### Methane diffusive flux

The methane diffusive flux was calculated based on sulfate concentration profiles in cores. The observed linear decreasing trend in sulfate profiles with depth suggests that sulfate reduction is driven primarily by methane consumption and, therefore, downward sulfate flux can be used as a proxy to an equal upward methane flux^40^. The flux was obtained by the Fick’s first law, and the effect of porosity on diffusion was considered through a logarithmic equation^63^. We assumed standard seawater salinity of 35 psu^64^ and sediment porosity of 0.6 (within the range of uncompacted mud^65^). The pressure and temperature for each piston core in the transect was obtained from bathymetric and CTD data, respectively (see Supplementary Data 2). The PC66 was not included in the flux calculation (and on the transect—Fig. 5) because its top was lost during core recovery.

### Gas chemical and isotopic composition

Samples for gas analyses in sediments were collected at every 1.5 m in the cores (ca. 100 cm^3^) and placed in gas-tight, inert jars, and kept at 4 °C. The sediment filled one third of the jar, while another third was filled with distilled water, leaving the top third with air (headspace). Five drops of the diluted Zephiran Chloride bactericide were added into each jar to eliminate microbial activity. Gas composition (seeping gas from plumes and headspace gas from jars) was determined with a gas chromatograph equipped with a capillary column VP-Plot Alumina/KCl, 30 m × 0.53 mm, and a flame ionisation detector (FID). Helium was used as the carrier gas at a constant rate of 5 mL min^−1^. The injecting temperature was 190 °C and the FID temperature was held at 200 °C. Carbon stable isotopic analyses of methane were performed using a Thermo Fisher Scientific gas chromatograph coupled to a Thermo Scientific DELTA-V Plus isotope ratio monitoring mass spectrometer via a Thermo GC IsoLink and Conflo IV interfaces (Thermo Fisher Scientific). The gas chromatograph contained a 30 m × 0.32 mm fused silica column, Carboxen Plot 1006, and was operated at a heating ramp of 70–150 °C, over 30 min. The isotopic data are reported using the delta notation (δ^13^C) in parts per thousand (‰) with isotopic ratio relative to the international Vienna Pee Dee Belemnite standard (V-PDB). The ^14^C analyses were performed using accelerator mass spectrometry in gas samples obtained from sediment pores, hydrate, and venting gas. The gas samples were injected in vacuum glass tubes (vacutainer) and send to Beta Analytic laboratory in Florida, U.S.A. The modern reference standard was 95% the ^14^C activity of the National Institute of Standards and Technology (NIST) Oxalic Acid (SRM 4990C) and calculated using the Libby carbon half-life (5568 years).

## Supplementary information

Supplementary Information

Peer Review File

Supplementary Data 1

Supplementary Data 2

Supplementary Data 3

Supplementary Data 4

Supplementary Movie

Description of Additional Supplementary Files

## Data Availability

All relevant data are included in the Supplementary material to this article.
