# Supplementary material for: Gas hydrate dissociation linked to contemporary ocean warming in the southern hemisphere
Source: Nat Commun. 2020 Jul 29;11:3788. doi: 10.1038/s41467-020-17289-z (PMC7391661; doi:10.1038/s41467-020-17289-z)
Supplement: Supplementary file 1 — Supplementary Information [file 41467_2020_17289_MOESM1_ESM.pdf]

Supplementary information for GAS HYDRATE DISSOCIATION LINKED TO CONTEMPORARY OCEAN WARMING IN THE SOUTHERN HEMISPHERE by KETZER et al.

Supplementary Note 1

Mass transfer rates and methane fluxes calculated for different bubble sizes and bubbling rates:

Bubble size 0.2 cm
0.023 bubbles per second
-mass transfer rate 4 10^{-6} g sec^{-1}
-advective mass flux 2 10^{-1} mmol cm^{-2} yr^{-1}
-methane transfer from sediments to the ocean in 0.25 $Mg.yr^{-1}$

0.106 bubbles per second
-mass transfer rate 1.85 10^{-5} g sec^{-1}
-advective mass flux 8.96 10^{-1} mmol cm^{-2} yr^{-1}
-methane transfer from sediments to the ocean 1.14 $Mg.yr^{-1}$

Bubble size 0.5 cm
0.023 bubbles per second
-mass transfer rate 6.21 10^{-5} g sec^{-1}
-advective mass flux 3 mmol cm^{-2} yr^{-1}
-methane transfer from sediments to the ocean 3.9 $Mg.yr^{-1}$

0.106 bubbles per second
-mass transfer rate 2.86 10^{-4} g sec^{-1}
-advective mass flux 13.87 mmol cm^{-2} yr^{-1}
-methane transfer from sediments to the ocean 17.8 $Mg.yr^{-1}$

Supplementary figure 1

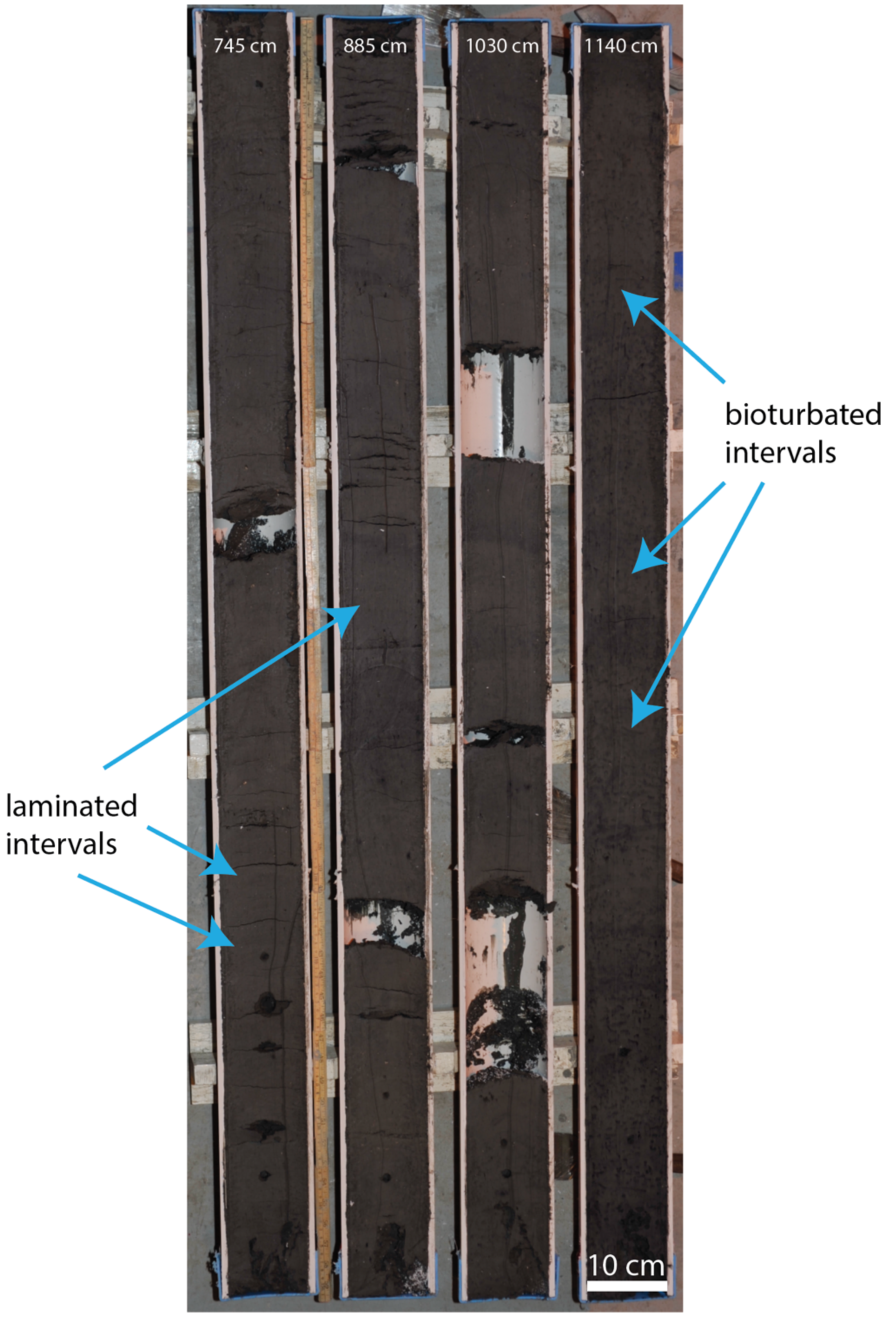

Supplementary figure 1 – Photograph of representative sediment core.

Photograph of four core sections from PC66 showing laminated and bioturbated intervals of dark olive and dark greenish grey muddy sediments. The numbers on the top of the figure are the depths below seafloor of each core section. See Figure 1 for core location.
