# Supplementary material for: Gas hydrate dissociation linked to contemporary ocean warming in the southern hemisphere
Source: Nat Commun. 2020 Jul 29;11:3788. doi: 10.1038/s41467-020-17289-z (PMC7391661; doi:10.1038/s41467-020-17289-z)
Supplement: Supplementary file 8 — Description of Additional Supplementary Files [file 41467_2020_17289_MOESM8_ESM.pdf]

Titles and captions for supplementary files for GAS HYDRATE DISSOCIATION LINKED TO CONTEMPORARY OCEAN WARMING IN THE SOUTHERN HEMISPHERE by KETZER et al.:

Supplementary Data 1 – Temperature and depth profiles.

Temperature and depth profiles obtained in the study area and used to calculate the depth of the feather edge of the methane hydrate stability zone.

Supplementary Data 2 – Sulphate concentration in porewaters.

Sulphate concentrations measured in porewater samples, and water depth and bottom water temperature obtained from bathymetric and CTD data, respectively for each of the six piston cores in the transect shown in Figure 5.

Supplementary Data 3 – Chemical composition of gas.

Chemical composition (C1 for methane, and C2+ for heavier hydrocarbons) of gas in venting bubbles, gas hydrate, and pore samples. Analyses with bdl are below detection limit. Samples marked with NA were not analysed.

Supplementary Data 4 – Isotopic composition of methane.

Isotopic ($\delta^{13}C$ and ^{14}C) analyses of methane in venting bubbles, hydrate and pore samples. The ^{14}C analyses are presented in percentage of modern carbon (pmC). Samples marked with NA were not analysed. For location refer to Figure 1.

Supplementary movie 1 – Bubble stream on the seafloor.

Bubble stream at flare site ROV08 (see Figure 1 for location).
