# Supplementary material for: Gas hydrate dissociation linked to contemporary ocean warming in the southern hemisphere
Source: Nat Commun. 2020 Jul 29;11:3788. doi: 10.1038/s41467-020-17289-z (PMC7391661; doi:10.1038/s41467-020-17289-z)
Supplement: Supplementary file 2 — Peer Review File [file 41467_2020_17289_MOESM2_ESM.docx]

Peer Review Information

**Journal:** Nature Communications

**Manuscript title:** Gas hydrate dissociation linked to contemporary ocean warming in the southern hemisphere

**Corresponding author name(s):** Prof Marcelo Ketzer

**Editorial notes:**

**Transferred manuscripts**

This manuscript has been previously reviewed at another journal that is not operating a transparent peer review scheme. This document only contains reviewer comments and rebuttal letters for versions considered at *Nature Communications*.

**Reviewer comments & decisions:**

**Reviewer comments, first version:**

Reviewer #1 (Remarks to the Author):

The authors present a new dataset from western South Atlantic at the Rio Grande Cone, presenting a large methane seepage area, and show that the ocean warming caused the gas hydrate stability zone to shift down slope in the region. Comparison between advective and diffusive flux indicates that the strong advective flux prevent the microbial community to consume methane before it reaches the seafloor. To this date, researchers consider that most methane is consumed in the sediment by sulphate reduction before it reaches the seafloor. This study seems to contradict this previous statement. The outcome of the study is interested and valuable for the community, however some statements are questionable as the reader lacks trivial information to reproduce the work. In addition, some material is omitted, making some statements and calculations questionable:

- How were the seepages located? Do they come from previous studies (in this case, citations are needed), or were they located during this present study (in this case, using what)? In the method, the authors claim that the bubbling sites were located during ROV dives, which is highly unlikely.
- Line 73 and figure 3: A figure of PC66 is shown to illustrate recovered gas hydrate. Why not showing a picture of a core illustrating the “massive and laminated, bioturbated, dark olive and dark greenish grey, rarely dark grey, muddy sediments containing authigenic carbonate nodules”?
- Line 86: The authors visited two sites with the ROV and based their calculation on observation from individual bubble streams at these two sites. However, they do not mention how many bubble streams they based their calculation upon, when we know that one site is most likely composed of several bubbles streams. As the observations were made for 1 hour, it is understandable that the focus was made on only one bubble stream. However, it would be valuable to indicate that many other bubbles streams were observed, but that the chosen bubble streams reflected the observations of each site. Most importantly, it is not possible to estimate a bubble size from the video presented in the supplementary material without any scale. One can therefore wonder how the average bubble size of 1 mm was estimated.
- Lines 88-91: The method section on gas quantification is too limited, and the calculation of mass transfer rates (not ratios at mentioned in the text, which has no unit), advective mass flux and annual rates are unclear, without any indication of exact location, temperature and depth. What area are we considering when converting these numbers to a yearly rate for the 394 flares? How were the numbers 28-130 Mg/yr calculated? Based on the mass transfer rates of 0.38e-3 and 1.74 e-3 g/sec, I get 5 and 22 Mg/year.
- Line 93 and figure 5: why is the transect including PC64 and not PC66, which is on the transect? If the measurements from PC66 are not usable, then the authors should mention why they did not use it.
- Lines 99-103: the statement explaining the decreasing methane concentration (not flux as mentioned in the manuscript) needs more references
Line 107-108: how were calculated the diffusive methane mass fluxes? The method description is too short and does give any information about the numbers used for the calculation. The authors refer to Figure 3, which does not illustrate the statement
- Line 136: Only 2 δ13C values are shown in PC93, with one value of -65.3 ‰ close to the δ13C values from other locations, and only one which is indeed highly depleted. Why are the two values from the same core so different? No depth is indicated. How about PC95 and PC97?
- Line 190: once again, how were the emission rates calculated? What section is taken into account here? What is its length?
- Supp Table 3 shows methane and ethane concentrations, both in ppm. Why not showing the mM conversion? Why omitting the sulphate concentration?

Other more specific comments:

- The manuscript is poorly written and needs editing from a native English speakers. Expressions such as “spectacular” or “world-class” should be avoided, as they sound more like a press release than a scientific article.
- One citation refers to a CD-rom, which I am not sure is adequate for this type of journals.
- The authors should indicate the panel they are referring to in a figure. The readers should not have to find the appropriate information themselves.
- Figure 1f: add CTD locations
- Line 88: Please refer to figure 3 and supp video
- Line 96: it is the depth of the maximum methane concentration that increases, not the maximum methane concentration that decreases downslope
- Line 99: which PC shows the 2.6m depth for the SMT? This information is not illustrated in Figure 5
- Line 99: why are we now talking about methane flux? Figure 5 only presents concentrations.
- Line 109: The maximum diffusive flux obtained by Egger et al. 2018 (inner shelf) is 0.87 mmol/m2/d, which is 31.8e-3 mmol/cm2/yr, i.e. 3 orders of magnitude more than the authors’ estimated advective flux (not 2 as written in the manuscript).
- Lines 109-112 unclear
- Line 146: the pockmark field is not represented in Figure 6 but Figure 1
- Line 158: I don’t think that Ruppel and Kessler 2016 mention that the upper slopes are depleted in methane
- Line 162-163: “base of the permanent thermocline lies in depths of 500-700 m” needs a reference
- Line 180: for people not working in the area, it might be useful to give a bit more information about AAIW (origin and T/S) in addition to the depth it is usually found
- Line 183: “within the depth range of most present-day gas flares” reads like this is the case world-wise. Add “on the RGC”
- Line 191-192: The numbers seem wrong. Sahling et al’s emission rates span from 2.4 to 6 x 103 mol/yr/m, corresponding to 2.4 - 6 x 106 mol/yr/km, not 0.3 – 5 x 106 mol/yr/km. Moreover, there is a mistake in this article and the conversion from volume flux (20.9 ml/min) to mass flux is 3727[kPa] * 0.0209[L/min] / (0.91 * 8.314 * 277.15 K) = 37.1 mmol/min and not 18.3 mmol/min for their area 3. This gives a total flow rate of 4.8 x 106 mol/yr/km and not 2.4 x 106 mol/yr/km in this area. You might consider using other references
- Supp Table 1: the authors present the entire profile for CTD67 (up and down), but only the down profile for the others.
- Supp Table 3: line 26, problem with reported depth


Reviewer #2 (Remarks to the Author):

Overview:

In this article the authors synthesize geological, geophysical, geochemical, and physical oceanographic observations collected from the Rio Grande Cone, on the southern Brazilian Atlantic margin to infer recent processes of methane hydrate dissociation and gas transport pathways in the subsurface. Specifically, multichannel seismic profile, piston core, CTD, ROV imagery, bathymetry (AUV and ship), acoustic backscatter (AUV and ship), and sub-bottom profile (SBP) (AUV) data were used to investigate subsurface methane dynamics proximal to the upper limit of the methane hydrate stability zone (MHSZ). Notably, the seismic data show a strong bottom simulating reflector (BSR) that outcrops upslope of the upper limit of the MHSZ. Additionally, spatially coincident high-resolution SBP profiles indicate vertical blanking beneath pockmarks and associated flares that were both imaged with a shipboard multibeam echosounder. Based upon these observations and a previously published rate for Antarctic intermediate water warming over the past 50 years, the authors conclude that anthropogenic warming has caused the upper limit of the MHSZ to move downslope away from the BSR outcrop resulting in the destabilization of the feather-edge hydrate, rapid vertical advective flux of dissociated gas, and contemporary discharge of methane to the ocean as observed water column flares. In addition to these observations, the authors also present results of isotopic analysis of discharged gas, which indicates that dissociated methane has a biogenic (shallow-microbial) origin. They additionally use the relative depth of the sulfur-methane transition zone observed in cores to quantify rates of diffusive methane flux and observation of bubble discharge rates and diameter to quantify advective methane flux.

General Comments:

A number of recent discoveries of continental slope methane discharge at the upper limit of the MHSZ suggest that this process is globally ubiquitous and demonstrate the relevance of methane hydrate dissociation and resultant advective flux as a critical terms in ocean carbon cycle budgets. Although the physics of hydrate stability are well understood, there are many open questions concerning characteristic time scales for hydrate dissociation, subsurface transport pathways for gas, and resultant carbon flux to the ocean. This article presents a suite of physical and chemical observations, from a relatively understudied region, that address these open questions, at least in part. Accordingly, it is a valuable contribution to the knowledge base of the field that will be welcomed by the gas hydrate and seep research communities. However, in some cases, the authors overstate the novelty of their results and their analysis is overly speculative. Additionally, there are some aspect of the presented research that would benefit from further analysis, greater clarity, and more thorough incorporation of relevant literature. Accordingly, it is my opinion that the article requires revision before it is suitable for publication in Nature Communications.

The major claim of the article is that anthropogenic warming of intermediate water can be causally linked to hydrate destabilization and resultant methane discharge from the seafloor. This claim is novel in a sense that the inferred process has not been previously observed in this region. However, the same process of anthropogenic warming resulting in MHSZ deepening away from the BSR outcrop as well as associated hydrate destabilization, dissociation, and seafloor gas discharge was previously reported on the Pacific Cascadia margin by Johnson et al. (2015) and modeled for the Arctic by Biatoch et al. (2011). Additionally, this claim could be strengthened and made more convincing by further supporting analysis of observations (detailed below). A secondary claim of this manuscript is that rapid dissociation of hydrate leads to vertical adventive methane flux through fractures directly to the ocean, making it inaccessible to anerobic oxidation in the subsurface. This claim is not particularly novel because SBP imaged blanking beneath pockmarks on the Rio Grande Cone of Brazil and the inference that the blanking represents vertical gas transport pathways from the BSR to the seafloor has previously been reported by Miller et al. (2015) and Rodrigues et al. (2017).

The claims in this article are not especially novel and I do not expect that they will substantially alter thinking in the field. However, the presented results do strengthen our understanding of the relationship between intermediate ocean warming and hydrate dissociation as well as associated methane transport pathways and rates. This is an important contribution that supports and corroborates previously proposed processes (i.e. Biastoch et al., 2011; Ferré and Feseker, 2012; Johnson et al., 2015) and is an important documentation of these phenomena in an understudied region. These results emphasize the global extent of these processes and provide a particularly comprehensive dataset that serves as good model for future data collection efforts on other margins. Specific suggestions for revisions to strengthen the manuscript are listed below.


Specific Comments:

Lines1-3: The title of the manuscript is overstated. Contemporary gas hydrate dissociation and related slope degassing has been previously recorded through direct observation at a number of locations in the southern hemisphere (e.g. Miller et al., 2015; Rodrigues et al, 2017; Greinert et al., 2010; Charlou et al., 2004; Faure et al., 2010; Davy et al., 2010; Ketzer et al., 2018; Ketzer et al., 2019).

Line 3: It is unclear why the word “Hemisphere” is capitalized.

Line 5-6: The first sentence of the abstract is overstated. It is not an accepted fact in the discipline that “Ocean warming related to contemporary climate change causes the dissociation of gas hydrate deposits and methane leakage on the seafloor.” In fact, I believe that is the hypothesis that the presented research is designed to address. If so, it should be presented as such in the abstract rather than stated as fact. I think it would be acceptable to state that “Ocean warming causes the dissociation of gas hydrate deposits and methane leakage on the seafloor.” Or “Ocean warming related to contemporary climate change has been proposed to cause the dissociation of gas hydrate deposits and methane leakage on the seafloor.”

Line 22: References 3 and 4 are rather dated. A more recent and thorough review of the relationship between seafloor methane hydrate and global climate is presented by Ruppel, 2011; and Ruppel and Kessler (2017) (manuscript references 5 and 18). Those documents indicate that continental margin hydrate has a relatively limited capacity to deliver methane to the atmosphere and regulate Earth’s climate. The authors acknowledge this later in lines 29-32. This paragraph could be clarified to better focus on current understanding in the discipline and indicate where specific further research is needed.

Line 27-29: Some of the refenced papers do not conclude or “argue” that anthropogenic-related ocean warming is causing hydrate dissociation. Specifically, refences 7 and 9 do not invoke anthropogenic warming as a driver of observed methane dissociation. Others listed references only suggest that it may be possible but do not provide direct supporting evidence.

Line 32: The authors may want to consider including a reference to the work of Kessler et al. (2011) which also demonstrated large scale oxidation of methane in the water column.

Line 48, 52, and 197: The terms “world-class” and “spectacular” are not particularly illuminating descriptors for a BSR outcrop. Consider providing a more technical description of why the outcrop is notable and useful for this analysis.

Line 62: I suggest the authors insert a reference that describes what “unit pockmarks” are, perhaps Hovland et al. (2010).

Lines 87-91: The calculations of advective flux make a number of assumptions and accordingly have a very high degree of uncertainty. For example, the calculations assume no temporal variability of advective flux on time scales longer than approximately one hour. Additionally, the calculations assume a uniform rate of discharge at all 394 flare sites. Furthermore, the calculations assume a uniform diameter of 1 cm for all bubbles. Previous long-term observation of advective gas discharge from other locations (e.g. Römer et al., 2012; Römer et al., 2016; Veloso et al., 2015) have shown that such assumptions are not reasonable. I understand that the authors were limited in these calculations by the available data. However, I think it is imperative that the authors explicitly acknowledge the substantial uncertainty in these calculations and the implications of that uncertainty (if any) for their conclusions.

Line 112-115: It is a bit speculative to assume that a fracturing process proposed by one numerical modeling study is the primary mechanism of advective gas transport. Does any of the collected subsurface profiles, seafloor survey data, or ROV imagery suggest fracturing? If not, the authors should consider referencing additional literature to further support their inferred mechanisms of advective gas transport.

Line 117-119: In reading the referenced paper (Skarke et al, 2014), it seems that an assumption that the majority of the methane released by hydrate dissociation in upper slope settings will be consumed anaerobically before reaching the seafloor is part of an ancillary calculation and not related to the fundamental results of that research. A better reference here would likely be Reeburgh (2007), which explicitly reports that ~88% of methane released from subsurface reservoirs is consumed by sulphate-coupled anaerobic oxidation of methane (AOM) in the sediment. In assessing the potential for anaerobic consumption of dissociated methane prior to discharge into the ocean, the authors should also consider the capacity of authigenic carbonate rock (which was widely observed at the field site) to capture large quantities of methane as detailed in Marlow et al. (2014).

Line 154-156: The wording here is a bit awkward. I suggest revision to clarify meaning.

Line 156: The paths of individual ocean currents is controlled by a myriad of factors. It is not reasonable to assume a “globally” consistent influx of warmer currents on continental shelves since the LGM. This sentence would be much improved if the word “currents” was changed to “intermediate waters” or even simply “water”

Line 156-159: The authors need to more clearly explain how upper slope hydrate dissociation driven by post-glacial warming could “possibly diminish the impact of short-term anthropogenic warming”

Line 159-174: Lines 159-167 contains an extended discussion of post -glacial cooling of the upper slope proposed by a single referenced article. It is immediately followed on lines 167-169 by quantitative evidence of upper slope warming that directly contradicts the basis of the preceding discussion on lines 159-167. The authors speculate that this could mean that the BSR has been stable for thousands of years but immediately concede on lines 173-174 that sufficient data to test that hypothesis is not available. This entire paragraph appears to lack a clear focus and does not seem to meaningfully contribute to the author’s primary claims. Significant revision of this paragraph for clarity is needed.

Lines 183-187 indicate that the edge of the MHSZ has moved from a depth of 530-540 m in the 1970s to a the present depth of 550-585 m, resulting in destabilization of hydrate and outgassing of dissociated methane in this interval. If that is the case, than outgassing in this interval should not have occurred prior to the 1970s, because it would have been within the MHSZ. However, on lines 143-149 the authors also report pockmarks with authigenic carbonate rock across the same depth interval (520-660m) “implying degassing over at least thousands of years.” This seem contradictory. The authors should clarify their inferred timeline of degassing, and MHSZ migration to address this apparent contradiction.

Line 402: The surname of the second author (Hornbach) has been omitted from this reference.

Line 413: The surname of the second author (Augustin) has been omitted from this reference.

Line 432: The surname of the second author (Kessler) has been omitted from this reference. Check all references for formatting error.

Figures:

Line 237: The caption for figure 1 states: “the core transect shown in Fig. 3 (yellow dashed line).” However, figure 3 does not present a core transect. Perhaps the authors meant figure 5?

Line 251: It would be useful for figure 2c to also indicate the depth range of observed seafloor pockmarks.


References cited in this review:

Johnson, H. P., Miller, U. K., Salmi, M. S., & Solomon, E. A. (2015). Analysis of bubble plume distributions to evaluate methane hydrate decomposition on the continental slope. Geochemistry, Geophysics, Geosystems, 16(11), 3825–3839. https://doi.org/10.1002/2015GC005955

Miller, D. J., Ketzer, J. M., Viana, A. R., Kowsmann, R. O., Freire, A. F. M., Oreiro, S. G., et al. (2015). Natural gas hydrates in the Rio Grande Cone (Brazil): A new province in the western South Atlantic. Marine and Petroleum Geology, 67, 187–196. https://doi.org/https://doi.org/10.1016/j.marpetgeo.2015.05.012

Rodrigues, L. F., Ketzer, J. M., Lourega, R. V., Augustin, A. H., Sbrissa, G., Miller, D., et al. (2017). The influence of methane fluxes on the sulfate/methane interface in sediments from the Rio Grande Cone Gas Hydrate Province, southern Brazil. Brazilian Journal of Geology, 47(3), 369–381. https://doi.org/10.1590/2317-4889201720170027

Greinert, J., Lewis, K. B., Bialas, J., Pecher, I. A., Rowden, A., Bowden, D. A., et al. (2010). Methane seepage along the Hikurangi Margin, New Zealand: Overview of studies in 2006 and 2007 and new evidence from visual, bathymetric and hydroacoustic investigations. Marine Geology, 272(1), 6–25. https://doi.org/https://doi.org/10.1016/j.margeo.2010.01.017

Charlou, J. L., Donval, J. P., Fouquet, Y., Ondreas, H., Knoery, J., Cochonat, P., et al. (2004). Physical and chemical characterization of gas hydrates and associated methane plumes in the Congo–Angola Basin. Chemical Geology, 205(3), 405–425. https://doi.org/https://doi.org/10.1016/j.chemgeo.2003.12.033

Faure, K., Greinert, J., von Deimling, J. S., McGinnis, D. F., Kipfer, R., & Linke, P. (2010). Methane seepage along the Hikurangi Margin of New Zealand: Geochemical and physical data from the water column, sea surface and atmosphere. Marine Geology, 272(1), 170–188. https://doi.org/https://doi.org/10.1016/j.margeo.2010.01.001

Davy, B., Pecher, I., Wood, R., Carter, L., & Gohl, K. (2010). Gas escape features off New Zealand: Evidence of massive release of methane from hydrates. Geophysical Research Letters, 37(21). https://doi.org/10.1029/2010GL045184

Ketzer, J. M., Augustin, A., Rodrigues, L. F., Oliveira, R., Praeg, D., Pivel, M. A. G., et al. (2018). Gas seeps and gas hydrates in the Amazon deep-sea fan. Geo-Marine Letters, 38(5), 429–438. https://doi.org/10.1007/s00367-018-0546-6

Ketzer, M., Praeg, D., Pivel, M. A. G., Augustin, A. H., Rodrigues, L. F., Viana, A. R., & Cupertino, J. A. (2019). Gas seeps at the edge of the gas hydrate stability zone on Brazil’s continental margin. Geosciences (Switzerland), 9(5), 1–11. https://doi.org/10.3390/geosciences9050193

Kessler, J. D., Valentine, D. L., Redmond, M. C., Du, M., Chan, E. W., Mendes, S. D., et al. (2011). A persistent oxygen anomaly reveals the fate of spilled methane in the deep Gulf of Mexico. Science (New York, N.Y.), 331(6015), 312–5. https://doi.org/10.1126/science.1199697

Hovland, M., Heggland, R., De Vries, M. H., & Tjelta, T. I. (2010). Unit-pockmarks and their potential significance for predicting fluid flow. Marine and Petroleum Geology, 27(6), 1190–1199. https://doi.org/https://doi.org/10.1016/j.marpetgeo.2010.02.005

Römer, M., Riedel, M., Scherwath, M., Heesemann, M., & Spence, G. D. (2016). Tidally controlled gas bubble emissions: A comprehensive study using long-term monitoring data from the NEPTUNE cabled observatory offshore Vancouver Island. Geochemistry, Geophysics, Geosystems, 17(9), 3797–3814. https://doi.org/10.1002/2016GC006528

Römer, M., Sahling, H., Pape, T., Bohrmann, G., & Spieß, V. (2012). Quantification of gas bubble emissions from submarine hydrocarbon seeps at the Makran continental margin (offshore Pakistan). Journal of Geophysical Research: Oceans, 117(10). https://doi.org/10.1029/2011JC007424

Veloso, M., Greinert, J., Mienert, J., & De Batist, M. (2015). A new methodology for quantifying bubble flow rates in deep water using splitbeam echosounders: Examples from the Arctic offshore NW-Svalbard. Limnology and Oceanography: Methods, 13(6), 267–287. https://doi.org/10.1002/lom3.10024

Reeburgh, W. S. (2007). Oceanic methane biogeochemistry. Chem. Rev. 107, 486–513

Marlow, J. J., Steele, J. A., Ziebis, W., Thurber, A. R., Levin, L. A., & Orphan, V. J. (2014). Carbonate-hosted methanotrophy represents an unrecognized methane sink in the deep sea. Nature Communications, 5, 1–12. https://doi.org/10.1038/ncomms6094


Reviewer #3 (Remarks to the Author):

Review of NCOMMS-20-09067-T, “First record of contemporary gas hydrate dissociation and related slope degassing in the southern Hemisphere”

This is an impressive paper building on a significant body of analyses. The paper appears to be a high-end, “capstone” paper presenting the highlights of a number of studies that have been or will be presented in more detail elsewhere. Results are exciting results and certainly warrant this approach. The results are all sound, with a few minor comments listed below. The analyses all appear reproducible, provided data are fully accessible.

I certainly would not object to this paper being published in Nature Comms. In fact, if the other reviewers were of that opinion, I’d fully support it. I myself however, am a bit torn. The core conclusions from this study, from a global perspective, read like a combination of Phrampus & Hornbach (2012) (effect of bottom-water temp. warming) and Westbrook et al. (2009) (flares right above BSR pinchouts). Just because this is observed in the southern hemisphere, does not make it groundbreaking beyond the Earth Sciences community. The most novel aspect, in a global sense, is the observation of the 14C anomaly (or lack therefore) but they are not really a core part of the "story".

A few minor comments:
• L. 88+: Advective vs diffusive gas fluxes: This extrapolation has a high possibility of a bias since measurements were probably taken at locations that were deemed interesting to begin with. However, it is probably as good as it gets – this should just be acknowledged.
• L. 105: It is great to see these calculations.
• L. 117-119: I am not convinced that it is the general view that the “majority” of methane is consumed before it reaches the seafloor. How would seep sites exist? In my view, this aspect of the results may be a bit overhyped. The real challenge is how much (if any) make it to the atmosphere and how does it affect ocean chemistry.
• L. 121+, Origin of gas: The lack of 14C is fascinating! I suspect/hope this will be analyzed in more detail and published in a different journal.
• L. 151: It appears to me that there is some debate whether temperatures in the study area are in- or decreasing. At least for a full-length paper in another journal, this discussion should be extended.
• L. 176+, Fig. 4: I would be careful with a potential overinterpretation of the phase diagram at this level of accuracy. Slight changes of gas composition may lead to small (but in this context, significant) shifts of the phase boundary. For example, have fractions of CO2 of N2 been measured? Also, the salinity effect may not be entirely accurate (e.g., compare Dickens & Quinby-Hunt to using 0.35% salinity in HYDOFF/CSMHYD).

Again, I am highly supportive of this sound and exciting study. I am just not sure it is of significantly broad appeal beyond the Earth Sciences community to be published in Nature Comms. I am more than happy to be overruled.

====
References:

Dickens, G. R., and Quinby-Hunt, M.-S., 1994, Methane hydrate stability in seawater: Geophys. Res. Lett., v. 21, p. 2115-2118.

Phrampus, B. J., and Hornbach, M. J., 2012, Recent changes to the Gulf Stream causing widespread gas hydrate dissociation: Nature, v. 490, no. 7421, p. 527.

Westbrook, G. K., Thatcher, K. E., Rohling, E. J., Piotrowski, A. M., HeikoPälike, H., Osborne, A. H., Nisbet, E. G., Minshull, T. A., Lanoisellé, M., James, R. H., Hühnerbach, V., Green, D., Fisher, R. E., Crocker, A. J., Chabert, A., Bolton, C., Beszczynska-Möller, A., Berndt, C., and Aquilina, A., 2009, Escape of methane gas from the seabed along the West Spitsbergen continental margin: Geophys. Res. Lett., v. 36, p. L15608.

**Author rebuttal, first version:**

**Reviewer #1** (Remarks to the Author):

The authors present a new dataset from western South Atlantic at the Rio Grande Cone, presenting a large methane seepage area, and show that the ocean warming caused the gas hydrate stability zone to shift down slope in the region. Comparison between advective and diffusive flux indicates that the strong advective flux prevent the microbial community to consume methane before it reaches the seafloor. To this date, researchers consider that most methane is consumed in the sediment by sulphate reduction before it reaches the seafloor. This study seems to contradict this previous statement. The outcome of the study is interested and valuable for the community, however some statements are questionable as the reader lacks trivial information to reproduce the work. In addition, some material is omitted, making some statements and calculations questionable:

- How were the seepages located? Do they come from previous studies (in this case, citations are needed), or were they located during this present study (in this case, using what)? In the method, the authors claim that the bubbling sites were located during ROV dives, which is highly unlikely.

- We agree that we need to provide a more detailed explanation about how the seeps were located. The following text was included in the *Methods - Acoustic surveys*: “**The major cluster of gas flares shown in Figure 1d were located using the water column backscatter data obtained from the hull-mounted multi-beam echo sounder installed onboard the Rig Supporter vessel. The water column backscatter data, which is highly sensitive to the presence of gas bubbles in water and, therefore, can be used to detect flares^54^, was integrated with the seafloor backscatter data using the IVS-3D Fledermaus software. Individual gas flares shown in Figure 1f were located using the high-resolution water column data obtained from the AUV-mounted multi-beam echo sounder and side-scan sonar systems. Two flares (marked in Figure 1f) were selected for ROV investigations. The ROV front sonar was used to reach the flares**”.

We added to following reference:

54. Zhao, J.M., J.; Zhang, H.; Wang, S., Comprehensive detection of gas plumes from multibeam water column images with minimisation of noise interferences. *Sensors*, 2017. 17.

- Line 73 and figure 3: A figure of PC66 is shown to illustrate recovered gas hydrate. Why not showing a picture of a core illustrating the “massive and laminated, bioturbated, dark olive and dark greenish grey, rarely dark grey, muddy sediments containing authigenic carbonate nodules”?

- We agree with the referee and decided to make another figure to show some of the aforementioned features. The new figure was included in the supplementary material (Supplementary figure 1).

- Line 86: The authors visited two sites with the ROV and based their calculation on observation from individual bubble streams at these two sites. However, they do not mention how many bubble streams they based their calculation upon, when we know that one site is most likely composed of several bubbles streams. As the observations were made for 1 hour, it is understandable that the focus was made on only one bubble stream. However, it would be valuable to indicate that many other bubbles streams were observed, but that the chosen bubble streams reflected the observations of each site. Most importantly, it is not possible to estimate a bubble size from the video presented in the supplementary material without any scale. One can therefore wonder how the average bubble size of 1 mm was estimated.

- We agree with the referee and added the information of number of bubble streams per flare. The modified sentence (in bold) is: “Within the area of gas flares, ROV seafloor observations at two sites (Fig. 1**f**) indicate that **acoustically-observed flares are formed by 5-6** individual bubble streams (each bubble ca. 1 cm diameter), **which** flow intermittently, varying from virtually zero to up to 3 bubbles per second in a time span of less than a minute **(Fig. 3a, Supplementary video)**. **Bubbles are estimated to be ca. 1 cm in diameter based on observations during sampling of a single bubble stream at each site (see Methods)**. Observations **of these two sites** over longer periods (up to 1 hour) yield mass transfer **rates** of…”
- The size of the bubbles was estimated using markings on the gas sampler (acrylic funnel). We added the following information on the existing sentence in the *Methods – Flare inspection, and gas sampling and quantification:* “…coupled to a ball valve and a transparent, acrylic funnel **with markings to estimate bubble size.**”

- Lines 88-91: The method section on gas quantification is too limited, and the calculation of mass transfer rates (not ratios at mentioned in the text, which has no unit), advective mass flux and annual rates are unclear, without any indication of exact location, temperature and depth. What area are we considering when converting these numbers to a yearly rate for the 394 flares? How were the numbers 28-130 Mg/yr calculated? Based on the mass transfer rates of 0.38e-3 and 1.74 e-3 g/sec, I get 5 and 22 Mg/year.

- We agree and have provided more details in the Method section. The values obtained by the referee are correct, but they should be multiplied by 5, as we assumed an average 5 bubble streams per flare (this information was missing in the first version, but has been updated now). We have also updated the compressibility factor for gas and obtained a slightly more precise values for our calculations (a small difference from the original values, but we think it is appropriated to make this update). Original values for mass transfer rates were 0.38x10^-3^ g/sec and 1.74x10^-3^ g/sec, and the new ones are 0.5x10^-3^ g/sec and 2.3x10^-3^ g/sec, respectively. The new related mass fluxes are 25.2 and 115.9 mmol cm^-2^ yr^-1^ (old ones were 26.6 and 106.5, respectively). The new total methane mass transfer rate from sediments to the ocean is 31.3 – 144 Mg/yr (old values are 28 and 130, respectively). The two areas inspected with ROV are shown in Fig. 1f, and the temperature and pressure used for calculations are now informed in the Methods section.

- Line 93 and figure 5: why is the transect including PC64 and not PC66, which is on the transect? If the measurements from PC66 are not usable, then the authors should mention why they did not use it.

- We agree and have provided an explanation of why PC66 was not used. We added the following sentence to the *Methods - Methane diffusive flux*: “**The PC66 was not included in the flux calculation (and on the transect - Fig. 5) because its top was lost during core recovery.**”

- Lines 99-103: the statement explaining the decreasing methane concentration (not flux as mentioned in the manuscript) needs more references

- We agree and have added the following reference:

Haacke, R.R., et al., Migration and venting of deep gases into the ocean through hydrate-choked chimneys offshore Korea. Geology, 2009. 37(6): p. 531-534.

Line 107-108: how were calculated the diffusive methane mass fluxes? The method description is too short and does give any information about the numbers used for the calculation. The authors refer to Figure 3, which does not illustrate the statement

- We agree and have provided more details in the Methods section, and included depth (pressure) and temperature values in the Supplementary table 2. In addition, we removed the methane solubility part because it was not relevant for the discussions in the paper. The following text has been added to the Methods: “**We assumed seawater salinity of 35psu and sediment porosity of 0.6. The pressure and temperature for each piston core in the transect was obtained from bathymetric and CTD data, respectively (see Supplementary table 2)**.”

- Line 136: Only 2 δ^13^C values are shown in PC93, with one value of -65.3 ‰ close to the δ13C values from other locations, and only one which is indeed highly depleted. Why are the two values from the same core so different? No depth is indicated. How about PC95 and PC97?

- We agree and have provided the required information (see Supplementary table 3). The highly depleted value (-80 ‰) is found at the middle of the core (1350 mbsl), while the less depleted value (-65.3 ‰) is found close to its base (2860 mbsl). The PC95 and PC97 don’t show highly depleted values because they are positioned inside the active venting area, while PC93 is away from it. We updated Supplementary table 3 with depths for all samples, including PC95 and PC97. We believe those points are now clearer.

- Line 190: once again, how were the emission rates calculated? What section is taken into account here? What is its length?

- We agree and details of the calculation have been added (see comments above for Lines 88-91 and 107-108). In addition, we have added the length of the studied section in the sentence on Lines 189-193 (bold): “It is interesting to note that the methane emission rates estimated for the **12 km long studied** **RGC** **section**…”.

- Supp Table 3 shows methane and ethane concentrations, both in ppm. Why not showing the mM conversion? Why omitting the sulphate concentration?

- We assume that the reviewer is referring to Table 2 (gas composition) rather than Table 3 (isotopes). We agree and the values in Table 2 are now in mM. In addition, we added a new table (Supplementary table 4) with sulphate data (mM).

Other more specific comments:

- The manuscript is poorly written and needs editing from a native English speakers. Expressions such as “spectacular” or “world-class” should be avoided, as they sound more like a press release than a scientific article.

- We agree and have removed the terms “spectacular” and “world-class”. The second author is a native speaker and has made now a careful revision of the manuscript.

- One citation refers to a CD-rom, which I am not sure is adequate for this type of journals.

- This is an important reference from a conference (Abstract volume). We corrected the reference and replaced “CD-ROM” with “Abstract volume”.

- The authors should indicate the panel they are referring to in a figure. The readers should not have to find the appropriate information themselves.

- We agree with the referee and we have changed the references to figures to include the relevant panels throughout the text according to this suggestion.

- Figure 1f: add CTD locations

- We have added CTDs locations to Figure 1f.

- Line 88: Please refer to figure 3 and supp video

- We have referred to Figure 3 and Supplementary video.

- Line 96: it is the depth of the maximum methane concentration that increases, not the maximum methane concentration that decreases downslope

- We agree and have modified the sentence (bold): “**Along** the transect, **the depth of the maximum** methane concentration **increases** downslope from the hydrate free area into the GHSZ…”.

- Line 99: which PC shows the 2.6m depth for the SMT? This information is not illustrated in Figure 5

- This was a mistake. The value should be 3.2m and not 2.6m as indicated in the text. This has been corrected.

- Line 99: why are we now talking about methane flux? Figure 5 only presents concentrations.

- We agree and have modified the sentence (bold): The **increasing depth of the SMT and maximum methane concentration, and related smaller** methane flux within the thickening GHSZ is attributed to a combination of the uptake of methane via hydrate precipitation…”.

- Line 109: The maximum diffusive flux obtained by Egger et al. 2018 (inner shelf) is 0.87 mmol/m2/d, which is 31.8e-3 mmol/cm2/yr, i.e. 3 orders of magnitude more than the authors’ estimated advective flux (not 2 as written in the manuscript).

- We thank the referee for pointing this out and have corrected the mistake. We have modified the text to make clear our results are comparable to those of Egger et al. (2018).

- Lines 109-112 unclear

- We agree with the referee and proposed a new sentence to replace the old one: “**It is interesting to note that this massive advective flux bypasses the local sulphate reduction zone without changing the local SMT depth (5.1m at PC64; Fig. 5), indicating it is very localised**.”

- Line 146: the pockmark field is not represented in Figure 6 but Figure 1

- We agree and have corrected this mistake.

- Line 158: I don’t think that Ruppel and Kessler 2016 mention that the upper slopes are depleted in methane

- We would argue that this information could be deduced from Ruppel and Kessler’s paper. However, this reference is not strictly necessary for the point we want to make, and so we have removed it from that sentence.

- Line 162-163: “base of the permanent thermocline lies in depths of 500-700 m” needs a reference

- We agree and have added a reference.

- Line 180: for people not working in the area, it might be useful to give a bit more information about AAIW (origin and T/S) in addition to the depth it is usually found

- We agree and have added the following sentence (with a reference): “**This water mass is formed near the Antarctic Circumpolar Current, flows at >500 m water depth at the RGC, and is characterized by a salinity minimum and dissolved oxygen maximum^49^**.”

Reference:

49.	Talley, L. D. in The South Atlantic: Present and Past Circulation (ed G.; Berger Wefer, W.H.; Siedler, G.; Webb, D.J.) 219-238 (Springer-Verlag, 1996).

- Line 183: “within the depth range of most present-day gas flares” reads like this is the case world-wise. Add “on the RGC”

- We agree and have added “**on the RGC**” at the end of the sentence.

- Line 191-192: The numbers seem wrong. Sahling et al’s emission rates span from 2.4 to 6 x 103 mol/yr/m, corresponding to 2.4 - 6 x 106 mol/yr/km, not 0.3 – 5 x 106 mol/yr/km. Moreover, there is a mistake in this article and the conversion from volume flux (20.9 ml/min) to mass flux is 3727[kPa] * 0.0209[L/min] / (0.91 * 8.314 * 277.15 K) = 37.1 mmol/min and not 18.3 mmol/min for their area 3. This gives a total flow rate of 4.8 x 106 mol/yr/km and not 2.4 x 106 mol/yr/km in this area. You might consider using other references

- We acknowledge the comment about Sahling et al.’s paper. In view of the mistake pointed out by the referee, we decided to remove the values from our manuscript. However, we have kept the reference because the original values and the corrected ones proposed by the referee are in the same order of magnitude and, therefore, don’t change our argument (comparison with the RGC). We followed the referee’s advice and added two other references (Westbrook et al., 2009, Reagan et al., 2011). These two references contain considerably higher values than the ones from the RGC and, therefore, we modified the last sentence of the paragraph (bold): “It is interesting to note that the methane emission rates estimated for the 12 km long studied RGC section (0.16 x 10^6^ to 0.75 x 10^6^ mol yr^-1^ km^-1^) are **smaller** **than** those of the western Svalbard margin^51-53^, suggesting a methane emission range of 10^5^ to 10**^7^** mol yr^−1^ km^-1^ for such settings.”

New references:

52. Westbrook, G. K., et al. Escape of methane gas from the seabed along the west Spitsbergen continental margin, Geophys. Res. Lett., 36, L15608, doi:10.1029/2009GL039191, 2009.

53. Reagan, M. T., et al. Contribution of oceanic gas hydrate dissociation to the formation of Arctic Ocean methane plumes, J. Geophys Res.-Oceans, 116, C09014, doi:10.1029/2011jc007189, 2011.

- Supp Table 1: the authors present the entire profile for CTD67 (up and down), but only the down profile for the others.

- We have corrected this and CTD67 contains now only the “down profile” values.

- Supp Table 3: line 26, problem with reported depth

- We have corrected this. Supplementary table 3 contains now the corrected depth.

Reviewer #2 (Remarks to the Author):

Overview:

General Comments:

A number of recent discoveries of continental slope methane discharge at the upper limit of the MHSZ suggest that this process is globally ubiquitous and demonstrate the relevance of methane hydrate dissociation and resultant advective flux as a critical terms in ocean carbon cycle budgets. Although the physics of hydrate stability are well understood, there are many open questions concerning characteristic time scales for hydrate dissociation, subsurface transport pathways for gas, and resultant carbon flux to the ocean. This article presents a suite of physical and chemical observations, from a relatively understudied region, that address these open questions, at least in part. Accordingly, it is a valuable contribution to the knowledge base of the field that will be welcomed by the gas hydrate and seep research communities. However, in some cases, the authors overstate the novelty of their results and their analysis is overly speculative. Additionally, there are some aspect of the presented research that would benefit from further analysis, greater clarity, and more thorough incorporation of relevant literature. Accordingly, it is my opinion that the article requires revision before it is suitable for publication in Nature Communications.

The major claim of the article is that anthropogenic warming of intermediate water can be causally linked to hydrate destabilization and resultant methane discharge from the seafloor. This claim is novel in a sense that the inferred process has not been previously observed in this region. However, the same process of anthropogenic warming resulting in MHSZ deepening away from the BSR outcrop as well as associated hydrate destabilization, dissociation, and seafloor gas discharge was previously reported on the Pacific Cascadia margin by Johnson et al. (2015) and modeled for the Arctic by Biatoch et al. (2011). Additionally, this claim could be strengthened and made more convincing by further supporting analysis of observations (detailed below). A secondary claim of this manuscript is that rapid dissociation of hydrate leads to vertical adventive methane flux through fractures directly to the ocean, making it inaccessible to anerobic oxidation in the subsurface. This claim is not particularly novel because SBP imaged blanking beneath pockmarks on the Rio Grande Cone of Brazil and the inference that the blanking represents vertical gas transport pathways from the BSR to the seafloor has previously been reported by Miller et al. (2015) and Rodrigues et al. (2017).

The claims in this article are not especially novel and I do not expect that they will substantially alter thinking in the field. However, the presented results do strengthen our understanding of the relationship between intermediate ocean warming and hydrate dissociation as well as associated methane transport pathways and rates. This is an important contribution that supports and corroborates previously proposed processes (i.e. Biastoch et al., 2011; Ferré and Feseker, 2012; Johnson et al., 2015) and is an important documentation of these phenomena in an understudied region. These results emphasize the global extent of these processes and provide a particularly comprehensive dataset that serves as good model for future data collection efforts on other margins. Specific suggestions for revisions to strengthen the manuscript are listed below.

- We appreciate the comments of the referee and have addressed them throughout, by clarifying the novelty of the results with reference to previous work and improving the analysis of observations, consistent with the specific comments below.

Specific Comments:

Lines1-3: The title of the manuscript is overstated. Contemporary gas hydrate dissociation and related slope degassing has been previously recorded through direct observation at a number of locations in the southern hemisphere (e.g. Miller et al., 2015; Rodrigues et al, 2017; Greinert et al., 2010; Charlou et al., 2004; Faure et al., 2010; Davy et al., 2010; Ketzer et al., 2018; Ketzer et al., 2019).

- We agree with the referee that the previous title of the paper (“First record of contemporary gas hydrate dissociation and related slope degassing in the southern Hemisphere”) was misleading in relation to previous studies. However, while most of the papers cited above report contemporary gas seeps, they do not demonstrate clearly that hydrate is dissociating at present-day, nor that this can be linked to ocean warming. We believe our paper is the first to present a robust data set that demonstrates hydrate dissociation linked to contemporary ocean warming in the southern hemisphere. We therefore proposed a modification of the title to: “**First record of gas hydrate dissociation linked to contemporary ocean warming in the southern hemisphere**”.

Line 3: It is unclear why the word “Hemisphere” is capitalized.

- This mistake was corrected.

Line 5-6: The first sentence of the abstract is overstated. It is not an accepted fact in the discipline that “Ocean warming related to contemporary climate change causes the dissociation of gas hydrate deposits and methane leakage on the seafloor.” In fact, I believe that is the hypothesis that the presented research is designed to address. If so, it should be presented as such in the abstract rather than stated as fact. I think it would be acceptable to state that “Ocean warming causes the dissociation of gas hydrate deposits and methane leakage on the seafloor.” Or “Ocean warming related to contemporary climate change has been proposed to cause the dissociation of gas hydrate deposits and methane leakage on the seafloor.”

- We agree with the referee and have changed the first sentence of the abstract to accommodate the suggestion and accord with the new title. The new sentence is: “**Ocean warming related to climate change has been proposed to cause the dissociation of gas hydrate deposits and methane leakage on the seafloor.**”

Line 22: References 3 and 4 are rather dated. A more recent and thorough review of the relationship between seafloor methane hydrate and global climate is presented by Ruppel, 2011; and Ruppel and Kessler (2017) (manuscript references 5 and 18). Those documents indicate that continental margin hydrate has a relatively limited capacity to deliver methane to the atmosphere and regulate Earth’s climate. The authors acknowledge this later in lines 29-32. This paragraph could be clarified to better focus on current understanding in the discipline and indicate where specific further research is needed.

- We agree with the referee and have added the two aforementioned references to the text. We have added the following sentence to accommodate the suggestion of the referee (bold): “Clearly, it is important to improve our understanding of the long- to short-term dynamics of the upper limits of gas hydrate systems on upper continental slopes **in relation to contemporary climate change, and the effectiveness of the sulphate reduction filter in preventing methane to reach the oceans.**”

Line 27-29: Some of the refenced papers do not conclude or “argue” that anthropogenic-related ocean warming is causing hydrate dissociation. Specifically, refences 7 and 9 do not invoke anthropogenic warming as a driver of observed methane dissociation. Others listed references only suggest that it may be possible but do not provide direct supporting evidence.

- We agree with the referee and replaced “argue” with “suggest”, and modified the sentence. The modified sentence is (bold): “Field observations and numerical modelling **suggest** that anthropogenic-related ocean warming **may be** causing hydrate dissociation in upper slope settings”.

Line 32: The authors may want to consider including a reference to the work of Kessler et al. (2011) which also demonstrated large scale oxidation of methane in the water column.

- We agree and have added the suggested reference.

Line 48, 52, and 197: The terms “world-class” and “spectacular” are not particularly illuminating descriptors for a BSR outcrop. Consider providing a more technical description of why the outcrop is notable and useful for this analysis.

- We agree with the referee and have removed those terms from the paper.

Line 62: I suggest the authors insert a reference that describes what “unit pockmarks” are, perhaps Hovland et al. (2010).

- We agree and have added the suggested reference.

Lines 87-91: The calculations of advective flux make a number of assumptions and accordingly have a very high degree of uncertainty. For example, the calculations assume no temporal variability of advective flux on time scales longer than approximately one hour. Additionally, the calculations assume a uniform rate of discharge at all 394 flare sites. Furthermore, the calculations assume a uniform diameter of 1 cm for all bubbles. Previous long-term observation of advective gas discharge from other locations (e.g. Römer et al., 2012; Römer et al., 2016; Veloso et al., 2015) have shown that such assumptions are not reasonable. I understand that the authors were limited in these calculations by the available data. However, I think it is imperative that the authors explicitly acknowledge the substantial uncertainty in these calculations and the implications of that uncertainty (if any) for their conclusions.

- We agree with the referee and have acknowledged the uncertainties in the main text and Methods. In the text, we modified the lines referred to say (bold): “**Assuming** these rates **apply** to all 394 flares mapped in the area yields a total methane transfer from sediments to the ocean of 31.3 – 144 Mg.yr^-1^.” We added the following sentence to the Methods: “**The calculations assume no temporal variability of advective flux on time scales longer than one hour, a uniform rate of discharge at all 394 flare sites, and a single diameter of 1 cm for all bubbles.**”

Line 112-115: It is a bit speculative to assume that a fracturing process proposed by one numerical modeling study is the primary mechanism of advective gas transport. Does any of the collected subsurface profiles, seafloor survey data, or ROV imagery suggest fracturing? If not, the authors should consider referencing additional literature to further support their inferred mechanisms of advective gas transport.

- We agree with the referee and added a sentence to emphasize that the primary mechanism of advective gas transport is poorly understood. None of the available data we have suggests fracturing. We added the following sentence: “**The mechanisms by which a focused methane flow migrates through the sediment to bypass the sulphate reduction filter remain poorly understood, but recent numerical modelling** …”.

Line 117-119: In reading the referenced paper (Skarke et al, 2014), it seems that an assumption that the majority of the methane released by hydrate dissociation in upper slope settings will be consumed anaerobically before reaching the seafloor is part of an ancillary calculation and not related to the fundamental results of that research. A better reference here would likely be Reeburgh (2007), which explicitly reports that ~88% of methane released from subsurface reservoirs is consumed by sulphate-coupled anaerobic oxidation of methane (AOM) in the sediment. In assessing the potential for anaerobic consumption of dissociated methane prior to discharge into the ocean, the authors should also consider the capacity of authigenic carbonate rock (which was widely observed at the field site) to capture large quantities of methane as detailed in Marlow et al. (2014).

- We agree with the referee and have added the suggested reference (Reeburgh, 2007). We have retained the reference to Skarke et al (2014) as it nonetheless supports our idea in this paragraph. The referee makes a good point about potential carbon uptake via carbonate precipitation. Most of the carbonates formed at those settings, however, are linked to the alkalinity generated as a byproduct of the anaerobic oxidation of methane via sulphate reduction, which has already been discussed.

Line 154-156: The wording here is a bit awkward. I suggest revision to clarify meaning.

- We agree with the referee and have simplified the sentence. The meaning is still the same and, therefore, we kept the references. We added “pressure increase on gas hydrate stability”. The new sentence is: “Globally, ocean warming since the LGM has counteracted the effect of rising sea levels **(pressure increase) on gas hydrate stability**, notably on upper continental slopes where the influx of warmer **water** resulted in downslope retreat of its feather edge^15,45^.”

Line 156: The paths of individual ocean currents is controlled by a myriad of factors. It is not reasonable to assume a “globally” consistent influx of warmer currents on continental shelves since the LGM. This sentence would be much improved if the word “currents” was changed to “intermediate waters” or even simply “water”

- We agree and have replaced “currents” with “water”.

Line 156-159: The authors need to more clearly explain how upper slope hydrate dissociation driven by post-glacial warming could “possibly diminish the impact of short-term anthropogenic warming”

- We agree with the referee and have changed the sentence to better explain the idea. The new sentence is: “**Post-glacial warming is argued to have driven widespread hydrate dissociation and degassing along continental margins, leading to the formation of a gas hydrate-depleted zone on upper slopes^46^. Such a natural, long-term depletion process may have diminished the quantity of gas hydrate available for dissociation related to short-term anthropogenic ocean warming^15^**.”

Line 159-174: Lines 159-167 contains an extended discussion of post -glacial cooling of the upper slope proposed by a single referenced article. It is immediately followed on lines 167-169 by quantitative evidence of upper slope warming that directly contradicts the basis of the preceding discussion on lines 159-167. The authors speculate that this could mean that the BSR has been stable for thousands of years but immediately concede on lines 173-174 that sufficient data to test that hypothesis is not available. This entire paragraph appears to lack a clear focus and does not seem to meaningfully contribute to the author’s primary claims. Significant revision of this paragraph for clarity is needed.

- We agree with the referee that the last part of this paragraph required improvement. We believe that this discussion is important for the manuscript, and have modified the text to make our point clearer. It was implicit that further studies, which are out of the scope of our paper, are necessary, and therefore, the last sentence of the paragraph was removed from the text. The new version is (bold): On the southern Brazilian margin, in contrast, it has been **hypothesised** that **elevation of the permanent thermocline during sea-level rise resulted in a** post-glacial cooling of the upper slope **that** suppressed hydrate dissociation and methane release^47^. The proposed mechanism **was suggested to be** relevant to the gas hydrate system of the RGC^47^, where the base of the permanent thermocline lies in depths of 500-700 m and so contains the upper limit of the GHSZ^29^. We note that long-term **post-glacial** cooling would have favoured gas accumulation and so could account for the presence of **a** well-developed BSR outcrop, **making the southern Brazilian margin a potential modern analogue to LGM margins**. **However,** constraints on deglacial temperature changes in the water column on the upper RGC **slope** record temperature increases of 3.5˚C in bottom waters for the interval 18-10 ka BP, with a possible peak of 6.6˚C at ca. 14 ka BP48. **The latter value is comparable to present-day bottom water temperatures, suggesting** a long-term (latest 14 ka) stabilisation of temperatures **that would have maintained the edge of the MHSZ near its** present-day depth. **Long-term stability of the MHSZ could in part account for a well-developed BSR outcrop in the RGC**.”

Lines 183-187 indicate that the edge of the MHSZ has moved from a depth of 530-540 m in the 1970s to a the present depth of 550-585 m, resulting in destabilization of hydrate and outgassing of dissociated methane in this interval. If that is the case, than outgassing in this interval should not have occurred prior to the 1970s, because it would have been within the MHSZ. However, on lines 143-149 the authors also report pockmarks with authigenic carbonate rock across the same depth interval (520-660m) “implying degassing over at least thousands of years.” This seem contradictory. The authors should clarify their inferred timeline of degassing, and MHSZ migration to address this apparent contradiction.

- We agree with the referee that the text required improvement to make our idea clearer. Our study shows that the edge of the MHSZ has been stable for a long period of time (possibly since 14 ka), allowing the formation of a well-defined BSR outcrop at 515-520 m. The original text gave the wrong idea that the presence of carbonate concretion was evidence of a system leaking gas for thousands of years. Palaeowater temperature reconstructions referred on the text (Chiessi et al., 2008), however, suggest stability of bottom temperature conditions and the hydrate system for thousands of years, with the preservation of the BSR outcrop at its present-day position. Our data indicates that this system is destabilized by contemporary ocean warming, causing hydrate dissociation and gas flaring. We propose the following changes in the text to clarify our idea:
- Lines 143-149 (bold): “The presence of carbonate concretions resulting from the anaerobic oxidation of methane^26^ implies **high gas flux** over timescales of thousands of years at this location^29^.”
- Lines 183-187 (bold): We suggest that recent ocean warming **has** **unbalanced** the **edge of a long-term, stable gas** hydrate system, **which** is in thermodynamic disequilibrium and undergoing dissociation **downslope of the BSR outcrop** (Fig. 6).

Reference:

Chiessi, C. M. M., S.; Paul, A.; Pätzold, J.; Groeneveld, J.; Wefer, G. South Atlantic interocean exchange as the trigger for the Bølling warm event. Geology 36, 919-922, doi:10.1130/G24979A.1 (2008).

Line 402: The surname of the second author (Hornbach) has been omitted from this reference.

- We corrected this mistake.

Line 413: The surname of the second author (Augustin) has been omitted from this reference.

- We corrected this mistake.

Line 432: The surname of the second author (Kessler) has been omitted from this reference. Check all references for formatting error.

- We corrected this mistake.

Figures:

Line 237: The caption for figure 1 states: “the core transect shown in Fig. 3 (yellow dashed line).” However, figure 3 does not present a core transect. Perhaps the authors meant figure 5?

- We corrected this mistake.

Line 251: It would be useful for figure 2c to also indicate the depth range of observed seafloor pockmarks.

- We have added this information to the captions.

Reviewer #3 (Remarks to the Author):

Review of NCOMMS-20-09067-T, “First record of contemporary gas hydrate dissociation and related slope degassing in the southern Hemisphere”

This is an impressive paper building on a significant body of analyses. The paper appears to be a high-end, “capstone” paper presenting the highlights of a number of studies that have been or will be presented in more detail elsewhere. Results are exciting results and certainly warrant this approach. The results are all sound, with a few minor comments listed below. The analyses all appear reproducible, provided data are fully accessible.

I certainly would not object to this paper being published in Nature Comms. In fact, if the other reviewers were of that opinion, I’d fully support it. I myself however, am a bit torn. The core conclusions from this study, from a global perspective, read like a combination of Phrampus & Hornbach (2012) (effect of bottom-water temp. warming) and Westbrook et al. (2009) (flares right above BSR pinchouts). Just because this is observed in the southern hemisphere, does not make it groundbreaking beyond the Earth Sciences community. The most novel aspect, in a global sense, is the observation of the 14C anomaly (or lack therefore) but they are not really a core part of the "story".

A few minor comments:

• L. 88+: Advective vs diffusive gas fluxes: This extrapolation has a high possibility of a bias since measurements were probably taken at locations that were deemed interesting to begin with. However, it is probably as good as it gets – this should just be acknowledged.

- We agree with the referee and have acknowledged the point by replacing the work “applying” with “assuming”.

• L. 105: It is great to see these calculations.

- Thanks!

• L. 117-119: I am not convinced that it is the general view that the “majority” of methane is consumed before it reaches the seafloor. How would seep sites exist? In my view, this aspect of the results may be a bit overhyped. The real challenge is how much (if any) make it to the atmosphere and how does it affect ocean chemistry.

- We agree with the referee that the most important challenge (from the perspective of climate change) is how much methane reaches the atmosphere. However, we already mentioned this in the final lines of the first paragraph following the abstract (having added an additional reference according to the suggestion of reviewer #2) and it would be repetitive to return to it here. We believe it is important to stress the idea that the majority of methane may not be consumed before leaking through the seafloor, because this is important for understanding the effects of ocean warming on gas hydrate stability. However, we decided to remove the word “common” in the last sentence of the abstract to accommodate the suggestion of the referee. The sentence is: “Under such conditions, methane is not fully accessible to anaerobic oxidation, challenging the assumption that it is mostly consumed by sulphate reduction before reaching the seafloor.”

• L. 121+, Origin of gas: The lack of 14C is fascinating! I suspect/hope this will be analyzed in more detail and published in a different journal.

- Yes, it is fascinating indeed. We hope to have other opportunities in the future to return to the area and collect more samples.

• L. 151: It appears to me that there is some debate whether temperatures in the study area are in- or decreasing. At least for a full-length paper in another journal, this discussion should be extended.

- We thank the referee for the note. In fact, our aim was to highlight the uncertainties in the area regarding long-term temperature changes, and this section has been modified according to the comments of reviewer #2 as well.

• L. 176+, Fig. 4: I would be careful with a potential overinterpretation of the phase diagram at this level of accuracy. Slight changes of gas composition may lead to small (but in this context, significant) shifts of the phase boundary. For example, have fractions of CO2 of N2 been measured? Also, the salinity effect may not be entirely accurate (e.g., compare Dickens & Quinby-Hunt to using 0.35% salinity in HYDOFF/CSMHYD).

- We thank the referee for the comment. This diagram is intended to illustrate the methane hydrate stability field in relation to the observed features (depth of piston cores, BSR outcrop, gas flares). To avoid potential overinterpretation, we have modified the captions of Figure 4 (bold): “Depth vs. temperature diagram **illustrating** the CTD profiles, the location of piston cores, BSR outcrop, depth range of gas flares and the depth range of the feather edge of the methane hydrate stability zone. The stability of methane hydrate was calculated **assuming** pure methane in seawater^34^.”

In addition to the comments above, we made the following changes to the figures:

1. Figure 1:

1a) “core transect in figure 3” was replaced with “core transect in figure 5”.

1e) and 1f) correction of graphic scales.

2. Figure 3:

The order of the photos was inverted to follow the referencing order on the text.

3. Figure 5:

Correction of graphic scales for methane concentration (mM).

4. Figure 6:

Correction of the methane flux value (leftmost blue arrow).

**Reviewer comments, second version:**

Reviewer #1 (Remarks to the Author):

Thank you to the authors for their responses to earlier comments from myself and other reviewers. The manuscript is improved; however, there are still several important aspects, mainly in terms of calculations needed to justify the discussion, that need clarification before publication in Nature Communications.

<i>Number of individual bubble streams (line 88):</i>
I still think that this sounds speculative: only two sites observed with 5-6 individual bubbles streams, leading to the assumption that all 394 flares will show the same trend. I suggest to add references, showing that this assumption is in agreement with other sites. For example:
- Average of 6 individual bubble streams per cluster offshore Svalbard: Sahling, H. et al. Gas emissions at the continental margin west of Svalbard: mapping, sampling, and quantification. Biogeosciences 11, 6029–6046 (2014).
- Between 3 and 10 individual bubble streams per cluster offshore Pakistan: Römer et al. Quantification of gas bubble emissions from submarine hydrocarbon seeps at the Makran continental margin (offshore Pakistan). JGR. Vol 117, C10015 (2012)
- 1 to 5 individual bubble streams per cluster at the NEPTUNE cabled observatory offshore Vancouver Island: Römer, M., Riedel, M., Scherwath, M., Heesemann, M., and Spence, G. D. (2016), Tidally controlled gas bubble emissions: A comprehensive study using long‐term monitoring data from the NEPTUNE cabled observatory offshore Vancouver Island, Geochem. Geophys. Geosyst., 17, 3797– 3814

<i>Bubble size estimate (line 91):</i>
Despite the precision that bubbles were “measured” using the funnel’s marking, this statement is not convincing; may be a picture of bubbles inside the funnel would help visualizing the bubbles, as one can question the distance of the bubble from the edge of the funnel that could lead to overestimation of the bubble size. Moreover, many publications found diameters of individual gas bubbles ~2 - 5 mm in average (e.g. Leifer and Culling, 2010; Römer et al. 2012; Veloso et al., 2015) and even several sizes within a cluster (Römer et al., 2012). I suggest to give a range of fluxes based on a more reasonable range of bubble sizes.
- Leifer, I., and D. Culling (2010), Formation of seep bubble plumes in the Coal Oil Point seep field, Geo Mar. Lett., 30, 339–353.
- Veloso, M., Greinert, J., Mienert, J. & De Batist, M. A new methodology for quantifying bubble flow rates in deep water using splitbeam echosounders: examples from the Arctic offshore NW-Svalbard. Limnol. Oceanogr. Methods 13, 267–287 (2015).
- Römer et al. 2012 same as above

<i>Gas quantification (lines 93 and methods line 359):</i>
I still have a problem with this calculation. Using the gas law n=PV/ZRT, with
P=5.25 MPa = 5.25e6 Pa,
V=(4/3) x π x (0,01/2)^3=5.24e-7m3 (for bubbles 1 cm in diameter)
Z=0.8702,
R= 8.3145 J/(mol K),
T=8.5°C=281.65K,
I obtain n=1.35e-3 mol.
Counting 16.04 grams of CH<sub>4</sub> in 1 mole, this gives us 2.16e<sup>-2</sup> g for one bubble. With 5 bubble streams and 3 bubbles per seconds per stream, this gives 3.25e<sup>-1</sup> g/sec and not 5e<sup>-4</sup> g/sec. Also, I obtain 8 mmol/cm2/y accounting for 5 bubble streams and 3 bubbles per seconds per stream and an area of 8000 m<sup>2</sup>. Something is missing in the text to reproduce the calculations. I am also unsure how the numbers 44 cm<sup>3</sup>/h and 200 cm<sup>3</sup>/h were calculated and why they are here.

<i>Annual mass transfer of methane (lines 95-96):</i>
Several studies mentioned the seasonal variability of methane release (e.g. Berndt et al., 2014; Sun et al., 2018; Ferré et al., 2020), as well as the spatial variability (Veloso et al., 2019, Ferré et al., 2020), implying that annual rates are highly overestimated when accounting for snap shot studies on a limited amount of flares. The authors should consider a paragraph discussing this variability to highlight the limitation of their findings.
- Berndt, C. et al. Temporal constraints on hydrate-controlled methane seepage off Svalbard. Science 343, 284–287 (2014).
- Sun, M.S. et al. Dissolved methane in the East China Sea: distribution, seasonal variation and emission. Mar. Chem., 202, pp. 12-26, 10.1016/j.marchem.2018.03.001 (2018)
- Ferré et al. Reduced methane seepage from Arctic sediments during cold bottom water conditions. Nature Geoscience, 1‐5. (2020)

<i>Diffusive flux (line 112 and method line 373):</i>
The calculation is once again still unclear. Why not adding the salinity data to the temperature profiles to justify the use of a salinity of 35? Where is the sediment porosity of 0.6 coming from?

<i>The authors omitted to answer one of my previous questions:</i>
Line 123 (now line 131): Table 2 (now supp. table 3) only shows pore water samples. Not hydrates and bubbles. It is therefore not possible to verify this statement (“Methane is the dominant gas present in all bubble, hydrate, and pore water samples”). How about CO<sub>2</sub> and C3?

<i>Other comments:</i>
Line 55: I am not sure of the necessity to add “to our knowledge unprecedentedly”. It makes the sentence heavy and overstated.
Line 62: At the seafloor. “The” is missing.
Line 63: Reveals (missing s)
Line 70: it is the first time the acronym MHSZ is used, as GHSZ was previously used. MHSZ comes back at several places in the manuscript. I suggest to keep GHSZ everywhere in the text.
Line 88: the parenthesis is now repetitive as the estimated diameter is details a few lines below
Line 106: are attributed
Line 223: Prevents methane from reaching the seafloor
Line 226: The last paragraph before the conclusion states that there is 1-2 orders of magnitude difference between the Brazilian margin and the Svalbard margin. This sentence in the conclusion says that it is comparable.
Caption in Figure 2: I don’t understand the part of the sentence “and as vertical columns above,”
Line 370: suggests (missing s)


Reviewer #2 (Remarks to the Author):

After reviewing the revised manuscript as well as the author’s point by point responses to comments from the previous round of review, I feel that all have been satisfactorily addressed.


Reviewer #3 (Remarks to the Author):

I did not have the time for a full re-review but mostly read the rebuttal letter, skim-read the paper, and checked a few details. In the interest of speed, my comments are based on that: Scientifically, the paper was sound to begin with so I do not have any problems with this paper being published. It’d be good to have some more information on bottom-water temperature changes (over 100s to 1000s of years) but if that information is not available, so be it.

It is up to the Editor to decide whether the paper is of sufficiently broad interest for Nature Comm. The core conclusions of this study, from a global perspective, are a combination of Phrampus & Hornbach (2012) (effect of bottom-water temp. warming) and Westbrook et al. (2009) (flares right above BSR pinchouts). The one fully novel (and really puzzling) aspect, in my view, the lack of 14C anomalies will be the topic of another paper.

Having said this, the results are new, sound, and I think, publishable in a high-impact journal. I am highly supportive of this sound and exciting study.

Ingo Pecher

References:
Phrampus, B. J., and Hornbach, M. J., 2012, Recent changes to the Gulf Stream causing widespread gas hydrate dissociation: Nature, v. 490, no. 7421, p. 527.
Westbrook, G. K., Thatcher, K. E., Rohling, E. J., Piotrowski, A. M., HeikoPälike, H., Osborne, A. H., Nisbet, E. G., Minshull, T. A., Lanoisellé, M., James, R. H., Hühnerbach, V., Green, D., Fisher, R. E., Crocker, A. J., Chabert, A., Bolton, C., Beszczynska-Möller, A., Berndt, C., and Aquilina, A., 2009, Escape of methane gas from the seabed along the West Spitsbergen continental margin: Geophys. Res. Lett., v. 36, p. L15608.

**Author rebuttal, second version:**

**Reviewer #1** (Remarks to the Author):

*Number of individual bubble streams (line 88):*

I still think that this sounds speculative: only two sites observed with 5-6 individual bubbles streams, leading to the assumption that all 394 flares will show the same trend. I suggest to add references, showing that this assumption is in agreement with other sites. For example:

- Average of 6 individual bubble streams per cluster offshore Svalbard: Sahling, H. et al. Gas emissions at the continental margin west of Svalbard: mapping, sampling, and quantification. Biogeosciences 11, 6029–6046 (2014).

- Between 3 and 10 individual bubble streams per cluster offshore Pakistan: Römer et al. Quantification of gas bubble emissions from submarine hydrocarbon seeps at the Makran continental margin (offshore Pakistan). JGR. Vol 117, C10015 (2012)

- 1 to 5 individual bubble streams per cluster at the NEPTUNE cabled observatory offshore Vancouver Island: Römer, M., Riedel, M., Scherwath, M., Heesemann, M., and Spence, G. D. (2016), Tidally controlled gas bubble emissions: A comprehensive study using long-term monitoring data from the NEPTUNE cabled observatory offshore Vancouver Island, Geochem. Geophys. Geosyst., 17, 3797– 3814

-	We agree with the referee. That is a good suggestion and we have added the three references. This is the new sentence (bold): “Assuming these rates apply to all 394 flares mapped in the area, and that each flare contains 5 bubble streams **(an assumption that is in agreement with observations of other flares offshore Svalbard^35^, Pakistan^36^, and Cascadia margin^37^)**, the total methane ...”.

*Bubble size estimate (line 91):*

Despite the precision that bubbles were “measured” using the funnel’s marking, this statement is not convincing; may be a picture of bubbles inside the funnel would help visualizing the bubbles, as one can question the distance of the bubble from the edge of the funnel that could lead to overestimation of the bubble size. Moreover, many publications found diameters of individual gas bubbles ~2 - 5 mm in average (e.g. Leifer and Culling, 2010; Römer et al. 2012; Veloso et al., 2015) and even several sizes within a cluster (Römer et al., 2012). I suggest to give a range of fluxes based on a more reasonable range of bubble sizes.

-	We agree with the referee and added the references for bubble sizes to account for a high variability of diameters. This is the new sentence (bold): “Bubbles are estimated to be ca. 1 cm in diameter based on observations during sampling of a single bubble stream at each site (see Methods), **but may show a high variability in diameter, even in the same cluster^37^, and commonly between 0.2-0.5 cm, as observed in other seep sites around the world^35-37^**.”

-	We also provided calculations for mass transfer rates and fluxes for bubbles with 2 and 5 mm in diameter as suggested by the referee. We decided to include these values in the Supplementary Material to avoid confusion with the many values already in the text. This is the new sentence (bold): “...yield mass transfer rates of 0.5 10^-3^ g sec^-1^ and 2.3 x 10^-3^ g

1 (5)

sec^-1^ **(for bubble of 1 cm in diameter; see Supplementary information for mass transfer rates and fluxes with other bubble sizes)**”.

*Gas quantification (lines 93 and methods line 359):*

I still have a problem with this calculation. Using the gas law n=PV/ZRT, with

P=5.25 MPa = 5.25e6 Pa,

V=(4/3) x π x (0,01/2)^3=5.24e-7m3 (for bubbles 1 cm in diameter)

Z=0.8702,

R= 8.3145 J/(mol K),

T=8.5°C=281.65K,

I obtain n=1.35e-3 mol.

Counting 16.04 grams of CH4 in 1 mole, this gives us 2.16e-2 g for one bubble. With 5 bubble streams and 3 bubbles per seconds per stream, this gives 3.25e-1 g/sec and not 5e-4 g/sec. Also, I obtain 8 mmol/cm2/y accounting for 5 bubble streams and 3 bubbles per seconds per stream and an area of 8000 m2. Something is missing in the text to reproduce the calculations. I am also unsure how the numbers 44 cm3/h and 200 cm3/h were calculated and why they are here.

-	We agree with the referee and modified the text to clarify our calculations and apologise for the misunderstanding. All values presented in the text are correct, but needed clarification to be reproduced. There is no need to change the values in the manuscript. We thank the referee for drawing our attention to the need for clarification of this point.

-	The pressure informed in the Methods was a direct conversion of the depth without considering the density of seawater. However, we used for the calculations the correct pressure of 5.294 MPa (instead of 5.25 MPa) equivalent 525 m of water depth. This gives a small difference in the mass of methane in each bubble: 2.16e^-2^ g (referee) and 2.18e^-2^ (manuscript). We updated the Methods section with the accurate value of 5.294 MPa (instead of 5.25 MPa). This is the new sentence (bold): “...where Z is the compressibility of methane (0.8702), P is pressure **(5.294 MPa at 525 m of water depth)**, V is the bubble volume...”. No need to change the main text.

-	The mismatching in the mass transfer rate calculations is related to the use of maximum bubbling rate (referee) and average bubbling rate (manuscript). The value of 3 bubbles per second is the maximum rate observed during one hour of observations. Using this maximum rate and 5 bubble streams, we will obtain the same value calculated by the referee: 3.25e^-1^ g/sec using the same pressure of P=5.25 MPa (using P=5.294 gives a similar value of 3.28e^-1^ g/sec). The average rates over one hour are 0.023 and 0.106 bubbles per second, or 44 cm^3^/h and 200 cm^3^/h. We preferred to use the average rates instead of the maximum rate and obtained 0.5e^-3^ g/sec and 2e^-3^ g/sec. To clarify this, we modified the sentence in the text (bold): “Observations of these two sites over longer periods (up to 1 hour) **show a lower bubbling rate of 0.023-0.106 bubbles per second (44-200 cm^3^/h) and a** yield mass transfer rates of 0.5 10^-3^ g sec^-1^ and 2.3 x 10^-3^ g sec^-1^...”.

-	The flux obtained by the referee (8 mmol/cm2/y) considers one flare with the maximum flow rate. We considered the average flow rate and all 394 flares. Our text was not clear about that. We reallocated this information in the text to make this point clear now: “Assuming these rates apply to all 394 flares mapped in the area, and that each flare contains 5 bubble streams (an assumption that is in agreement with observations of other flares offshore Svalbard^38^, Pakistan^37^, and Cascadia margin^39^), **the total methane advective mass flux is 25.2-115.9 mmol cm^-2^ yr^-1^**, and the total methane mass transfer rate from sediments to the ocean is 31.3 – 144 Mg.yr^-1^”.

2 (5)

*Annual mass transfer of methane (lines 95-96):*

Several studies mentioned the seasonal variability of methane release (e.g. Berndt et al., 2014; Sun et al., 2018; Ferré et al., 2020), as well as the spatial variability (Veloso et al., 2019, Ferré et al., 2020), implying that annual rates are highly overestimated when accounting for snap shot studies on a limited amount of flares. The authors should consider a paragraph discussing this variability to highlight the limitation of their findings.

-	We agree with the referee and added three references for seasonal variability. We found opportune to add this limitation in the Methods section, where we have previously included other limitations of the calculations (previous revised version). The sentence in the Methods is (bold): “The calculations assume no temporal variability of advective flux on time scales longer than one hour, **no seasonal variations in the flux as observed in other seep sites^60-62^,** a uniform rate of discharge at all 394 flare sites, and a single bubble diameter of 1 cm for all bubbles **(see Supplementary information for mass transfer rates and fluxes with other bubble sizes)**”. The three suggested references were added to the above sentence.

*Diffusive flux (line 112 and method line 373):*

The calculation is once again still unclear. Why not adding the salinity data to the temperature profiles to justify the use of a salinity of 35? Where is the sediment porosity of 0.6 coming from?

-	We prefer to provide a reference for the salinity of 35psu (standard seawater salinity, Millero et al., 2008). We believe it would be unnecessary to add salinity values from CTD for that reason only.

-	The sediment porosity of 0.6 was assumed. This value is in the range of uncompacted muds (Mondol et al., 2007). We added this information and the reference in the Methods.

-	The new sentence is (bold): “We assumed **standard** seawater salinity of 35psu^64^ and sediment porosity of 0.6 **(within the range of uncompacted mud^65^)**”.

References:

Millero, F. J., Feistel, R., Wright, D. G. & McDougall, T. J. The composition of Standard Seawater and the definition of the Reference-Composition Salinity Scale. Deep Sea Research Part I: Oceanographic Research Papers 55, 50-72, doi:10.1016/j.dsr.2007.10.001 (2008).

Mondol, N. H., Bjørlykke, K., Jahren, J. & Høeg, K. Experimental mechanical compaction of clay mineral aggregates—Changes in physical properties of mudstones during burial. Marine and Petroleum Geology 24, 289-311, doi:10.1016/j.marpetgeo.2007.03.006 (2007).

The authors omitted to answer one of my previous questions:

Line 123 (now line 131): Table 2 (now supp. table 3) only shows pore water samples. Not hydrates and bubbles. It is therefore not possible to verify this statement (“Methane is the dominant gas present in all bubble, hydrate, and pore water samples”). How about CO2 and C3?

-	We apologise for the mistake. We now updated the Suppl. Table 3 with the gas

composition for gas hydrate and vent gas (including available CO2 analyses for pore gas). All hydrocarbons heavier than ethane are included in the same column in the table (as C2+).

Other comments:

Line 55: I am not sure of the necessity to add “to our knowledge unprecedentedly”. It makes the sentence heavy and overstated.

-	We agree with the referee and replaced “unprecedentedly” with “for the first time”.

3 (5)

Line 62: At the seafloor. “The” is missing.

- OK.

Line 63: Reveals (missing s)

- OK.

Line 70: it is the first time the acronym MHSZ is used, as GHSZ was previously used. MHSZ comes back at several places in the manuscript. I suggest to keep GHSZ everywhere in the text.

-	We agree and replaced all MHSZ with GHSZ.

Line 88: the parenthesis is now repetitive as the estimated diameter is details a few lines below

- OK.

Line 106: are attributed

- OK.

Line 223: Prevents methane from reaching the seafloor

- OK.

Line 226: The last paragraph before the conclusion states that there is 1-2 orders of magnitude difference between the Brazilian margin and the Svalbard margin. This sentence in the conclusion says that it is comparable.

-	We agree and replaced “comparable” with “lower than”.

Caption in Figure 2: I don’t understand the part of the sentence “and as vertical columns above,”

-	We agree that this part was unclear and removed it.

Line 370: suggests (missing s)

- OK.

-	We thank referee #1 for the valuable comments sent during the revisions of the manuscript.

Reviewer #2 (Remarks to the Author):

After reviewing the revised manuscript as well as the author’s point by point responses to comments from the previous round of review, I feel that all have been satisfactorily addressed.

-	We thank referee #2 for the valuable comments sent during the first revision of the manuscript.

Reviewer #3 (Remarks to the Author):

I did not have the time for a full re-review but mostly read the rebuttal letter, skim-read the paper, and checked a few details. In the interest of speed, my comments are based on that: Scientifically, the paper was sound to begin with so I do not have any problems with this paper being published. It’d be good to have some more information on bottom-water temperature changes (over 100s to 1000s of years) but if that information is not available, so be it.

It is up to the Editor to decide whether the paper is of sufficiently broad interest for Nature Comm. The core conclusions of this study, from a global perspective, are a combination of Phrampus & Hornbach (2012) (effect of bottom-water temp. warming) and Westbrook et al. (2009) (flares right

4 (5)

above BSR pinchouts). The one fully novel (and really puzzling) aspect, in my view, the lack of 14C anomalies will be the topic of another paper.

Having said this, the results are new, sound, and I think, publishable in a high-impact journal. I am highly supportive of this sound and exciting study.

References:

Phrampus, B. J., and Hornbach, M. J., 2012, Recent changes to the Gulf Stream causing widespread gas hydrate dissociation: Nature, v. 490, no. 7421, p. 527.

Westbrook, G. K., Thatcher, K. E., Rohling, E. J., Piotrowski, A. M., HeikoPälike, H., Osborne, A. H., Nisbet, E. G., Minshull, T. A., Lanoisellé, M., James, R. H., Hühnerbach, V., Green, D., Fisher, R. E., Crocker, A. J., Chabert, A., Bolton, C., Beszczynska-Möller, A., Berndt, C., and Aquilina, A., 2009, Escape of methane gas from the seabed along the West Spitsbergen continental margin: Geophys. Res. Lett., v. 36, p. L1560

-	We thank referee #3 for the valuable comments sent during the first revision of the manuscript

**Reviewer comments, third version:**

Reviewer #1 (Remarks to the Author):

I think the manuscript is much improved since the first version as it now includes all necessary steps to understand the conclusions. I support its publication in Nature Communications. My only comment is the format of references that should be carefully checked as many of them include typos.

**Author rebuttal, third version:**
